# Identification of a STIM1 Splicing Variant that Promotes Glioblastoma Growth

**DOI:** 10.1002/advs.202103940

**Published:** 2022-01-25

**Authors:** Jiansheng Xie, Guolin Ma, Lijuan Zhou, Lian He, Zhao Zhang, Peng Tan, Zixian Huang, Shaohai Fang, Tianlu Wang, Yi‐Tsang Lee, Shufan Wen, Stefan Siwko, Liuqing Wang, Jindou Liu, Yangchun Du, Ningxia Zhang, Xiaoxuan Liu, Leng Han, Yun Huang, Rui Wang, Youjun Wang, Yubin Zhou, Weidong Han

**Affiliations:** ^1^ Department of Medical Oncology Laboratory of Cancer Biology Institute of Clinical Science Sir Run Run Shaw Hospital College of Medicine Zhejiang University Hangzhou Zhejiang P. R. China; ^2^ Center for Translational Cancer Research Institute of Biosciences and Technology Texas A&M University Houston TX 77030 USA; ^3^ Beijing Key Laboratory of Gene Resource and Molecular Development College of Life Sciences Beijing Normal University Beijing 100875 P. R. China; ^4^ MOE Key Laboratory of Metabolism and Molecular Medicine Department of Biochemistry and Molecular Biology School of Basic Medical Sciences Fudan University Shanghai China; ^5^ Department of Biochemistry and Molecular Biology University of Texas Health Science Center at Houston McGovern Medical School Houston TX 77030 USA; ^6^ Center for Epigenetics and Disease Prevention Institute of Biosciences and Technology Texas A&M University Houston TX 77030 USA; ^7^ Department of Translational Medical Sciences College of Medicine Texas A&M University Houston TX 77030 USA

**Keywords:** calcium signaling, cell signaling, glioblastoma, splicing, STIM1

## Abstract

Deregulated store‐operated calcium entry (SOCE) mediated by aberrant STIM1‐ORAI1 signaling is closely implicated in cancer initiation and progression. Here the authors report the identification of an alternatively spliced variant of STIM1, designated STIM1β, that harbors an extra exon to encode 31 additional amino acids in the cytoplasmic domain. STIM1β, highly conserved in mammals, is aberrantly upregulated in glioma tissues to perturb Ca^2+^ signaling. At the molecular level, the 31‐residue insertion destabilizes STIM1β by perturbing its cytosolic inhibitory domain and accelerating its activation kinetics to efficiently engage and gate ORAI calcium channels. Functionally, STIM1β depletion affects SOCE in glioblastoma cells, suppresses tumor cell proliferation and growth both in vitro and in vivo. Collectively, their study establishes a splicing variant‐specific tumor‐promoting role of STIM1β that can be potentially targeted for glioblastoma intervention.

## Graphical abstract

The authors report a novel splice variant of stromal interaction molecule 1, designated STIM1β, that is abnormally upregulated in glioma cells. A 31‐residue insertion in the cytosolic domain of STIM1β renders itself prone for activation, thereby perturbing Ca^2+^ homeostasis and promoting glioblastoma growth. Their study establishes a splicing variant‐specific tumor‐promoting role of STIM1β that can be targeted for cancer intervention.

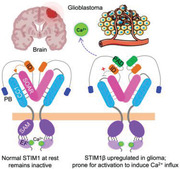

## 1 Introduction

Calcium (Ca^2+^) as a versatile intracellular secondary messenger plays an important role in regulating a diverse array of cellular processes such as gene expression, neurotransmitter release, muscle contraction, cell proliferation, differentiation, motility, and cell death.^[^ ^1^ ^]^ Store‐operated Ca^2+^ entry (SOCE) is regarded as a highly‐selective Ca^2+^ entry mechanism in both non‐excitable and excitable tissues in mammals.^[^ ^1^ ^]^ The agonist triggered depletion of the endoplasmic reticulum (ER) Ca^2+^ store is primarily detected by an ER‐resident Ca^2+^ sensor, stromal interaction molecule 1 (STIM1), which undergoes conformational changes and oligomerization to ultimately engage and activate the plasma membrane (PM)‐resident ORAI Ca^2+^ channels at the ER‐PM junctions, thereby permitting extracellular Ca^2+^ entry into the cytoplasm and subsequently refilling the ER Ca^2+^ store.^[^ ^1^, ^2^ ^]^ At least one splicing variant of STIM1 (STIM1L) and two variants of STIM2 (STIM2.2 and STIM2.3) have been identified, with STIM1L playing a tissue‐specific role in myocytes with faster SOCE activation whereas STIM2.2 and STIM2.3 serve as negative regulators of STIM.^[^ ^3^ ^]^ The existence of two STIM and three ORAI isoforms, as well as the splicing variants of STIM1/2 and ORAI1‐3,^[^ ^3^, ^4^ ^]^ has greatly contributed to the functional diversity of SOCE in different tissues under varying physiological conditions. The various STIM‐ORAI combinations lead to varying magnitudes of Ca^2+^ signals with distinct spatiotemporal features and different kinetics.^[^ ^1^, ^3^ ^]^ Aberrant SOCE signaling has been implicated in a growing number of diseases, such as immunodeficiency, cardiovascular disease, diabetes, and cancer.^[^ ^5^ ^]^ Thus, it is of great importance to explore what STIM‐ORAI combinations form SOC subtypes for each cell type and which ones contribute to Ca^2+^ entry in health and disease.

SOCE mediated by ORAI‐STIM signaling is among the major Ca^2+^ entry pathways in most types of cancer cells.^[^ ^6^ ^]^ Accumulating evidence supports a critical role of SOCE in cancer cell proliferation, metastasis, tumor microenvironment (TME) remodeling, and antitumor immunity.^[^ ^7^ ^]^ Altered expression of ORAI/STIM variants can be an early event in cancer development. Augmented SOCE arising from abnormal ORAI and/or STIM expression has been widely noted in breast, colorectal, cervical, liver, lung, and brain cancers.^[^ ^7^ ^]^ Conversely, pharmacological inhibition of SOCE and genetic depletion of ORAI/STIM have shown promising anti‐tumor effects by suppressing cancer cell migration and curtailing the growth of xenografts in mouse tumor models.^[^ ^7^ ^]^ Moreover, SOCE mediated Ca^2+^ signaling also plays an important role in TME remodeling. For example, STIM1 is reported to interact with hypoxia‐inducible factor‐1 (HIF1) and mediate hypoxia‐driven hepatocarcinogenesis.^[^ ^8^ ^]^ Clearly, aberrant SOCE is tightly linked to cancer growth, proliferation, migration, and metastasis.^[^ ^6^, ^7^ ^]^ Therefore, STIM and ORAI proteins promise to serve as excellent targets for developing anti‐cancer therapeutics. The SOCE‐associated Ca^2+^/calcineurin/nuclear factor of activated T‐cells (NFAT) pathway is a validated druggable target, as best exemplified by calcineurin inhibitors (e.g., tacrolimus and cyclosporine A), which are widely used in the clinical setting as an immunosuppressive agent to prevent graft rejection during organ transplantation.^[^ ^9^ ^]^ Similarly, pan‐ORAI/STIM inhibition is anticipated to impose a global immunosuppressive effect to dampen anti‐tumor immunity.^[^ ^10^ ^]^ It remains, therefore, challenging to balance the trade‐off between tumor‐inhibitory effects and immunosuppressive activities when unselectively targeting SOCE in both normal and cancer tissues. The discovery of STIM/ORAI variants that are differentially expressed in cancer tissues might overcome this issue by providing more selective targets for anti‐cancer therapeutic development.

Glioblastoma (GBM) is an aggressive malignant tumor due to its heterogeneity and plasticity.^[^ ^11^ ^]^ SOCE is essential for Ca^2+^ signaling and affects glioblastoma cell migration and invasion.^[^ ^12^ ^]^ Higher mRNA levels of STIM/ORAI genes were observed in primary GBM compared with human primary astrocyte cells.^[^ ^13^ ^]^ Silencing of STIM1 reduced the growth of glioma formation in a mouse xenograft model, emphasizing the potential oncogenic role of STIM1 in GBM.^[^ ^14^ ^]^ In the current study, we report the identification and functional characterization of a splicing variant of STIM1 (designated STIM1β), the expression of which is highly upregulated in glioblastoma and several other types of brain tumors. STIM1β harbors one extra exon (encoding 31 additional amino acids; designated as PAD for pro‐activation domain) in the cytoplasmic domain of STIM1 (STIM1‐CT), and functions as a potent ORAI1 channel activator following store depletion. We further demonstrate that the genetic depletion of STIM1β perturbed SOCE and pronouncedly curtailed the growth of glioblastoma cells both in vitro and in vivo, suggesting STIM1β as a promising selective therapeutic target for future brain cancer intervention.

## 2 Results

### 2.1 Discovery of a STIM1 Splicing Variant that Is Abnormally Upregulated in Certain Types of Cancers

**Figure 1:**
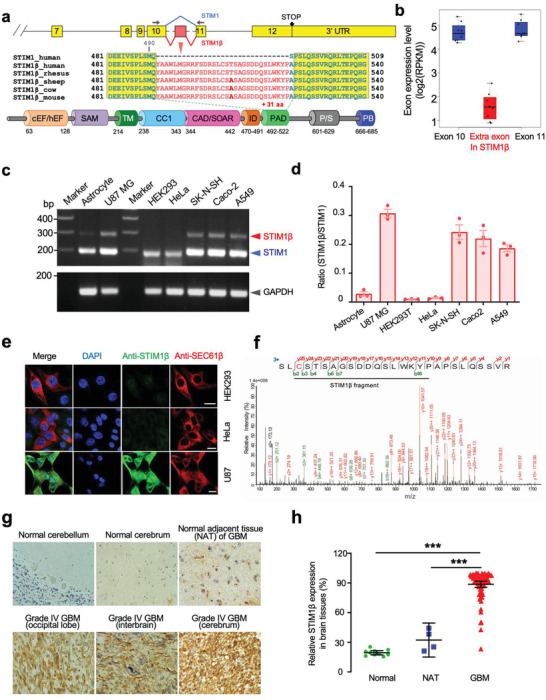
Discovery of STIM1β as a STIM1 alternative splicing variant that is upregulated in certain types of cancer. Data were shown as mean ± sem. a) Schematic of exon boundaries and alternative splicing of a previously‐unrecognized STIM1 variant (STIM1β). The primer pairs used to amplify the splice variant STIM1β or conventional STIM1 are shown as arrows. cEF/hEF, canonical or hidden EF‐hand; SAM, sterile alpha‐motif; TM, transmembrane domain; CC1, coiled coil region 1; CAD/SOAR, the STIM ORAI‐activating region; ID, inhibitory domain; PAD, pro‐activation domain; P/S, proline/serine‐rich region; PB, polybasic tail. b) The expression abundance of exon 10, the extra exon found in STIM1β (red), and exon 11 revealed by RNA‐seq. Boxplots denote expression distribution of each exon and each dot denotes RNA‐seq signals from one dataset. c,d) Comparison of the mRNA levels of STIM1 and STIM1β splice variants in selected cells by RT‐PCR. c) Agarose gel electrophoresis of PCR amplification of the region between exon 10 and exon 11. d) The relative expression ratio of STIM1β over STIM1 in the indicated cells. e) Immunostaining of STIM1β (green) in selected cell lines using a home‐made antibody specifically against STIM1β. Red, SEC61β as an ER marker. Scale bar, 10 µm. f) Detection of STIM1β protein in U87 cells with mass spectrometry. The polypeptide region containing PAD (underlined) was selected to perform MS/MS analysis to characterize its sequence identity. Assigned b_n_/y_n_ fragments were listed with the corresponding peptide sequences. An anti‐STIM1β antibody was used to enrich STIM1β from cell lysates. g) IHC staining of STIM1β in normal, normal adjacent tissues of tumor (NAT), and cancerous tissues from patients with brain tumors. h) Quantification of STIM1β expression in patient samples shown in panel (g) (Normal, *n* = 10; NAT, *n* = 4; GBM, *n* = 80). Also see Figure S3, Supporting Information.

We recently conducted a high throughput screen of small molecule modulators of the Ca^2+^/calcineurin‐NFAT pathway in U87 glioblastoma cells.^[^ ^15^ ^]^ During our analysis of the RNA‐seq data (GSE108749),^[^ ^15^ ^]^ we serendipitously discovered an extra exon that encodes 31 additional amino acids after position 491 (named as PAD for pro‐activation domain) in STIM1 (**Figure** **1**a). The abundance of this extra exon, judging from the RNA‐seq read density (Log_2_RPKM), was determined to be approximately 1/3 of the two neighboring exons 10 and 11 (Figure 1b). BLAST analysis further revealed that this STIM1 alternative splice variant, named as STIM1β, was different from the commonly‐spliced form of STIM1, and it remained well conserved among mammals (Figure 1a). To independently validate this finding, we designed two pairs of splice variant‐specific primers to compare the expression levels of conventional STIM1 and STIM1β side‐by‐side in human tissues by quantitative real‐time PCR (qRT‐PCR) (Figure S1a, Supporting Information). The mRNA expression of STIM1β was detected in most tissues but at a substantially lower level compared to STIM1 expression (Figure S1a, Supporting Information). Most notably, unlike STIM1 that is abundantly expressed in the hematopoietic and immune systems, STIM1β expression in lymphoid tissues and cells of the immune system turned out to be extremely low, with the STIM1β‐over‐STIM1 ratio ranging from 0.02 to 0.08 (Figure S1a, Supporting Information). Next, we examined STIM1β expression at the transcription level in both non‐cancerous human cells and cancer cell lines derived from various human tissues with qRT‐PCR (Figure 1c). A higher ratio of STIM1β/STIM1 (0.2–0.3 compared to 0.01–0.03 in astrocytes and non‐cancerous HEK293 cells) was noted in several cancer cell lines (Figure 1d), including U87 MG, SK‐N‐SH, Caco‐2, and A549 originated from glioblastoma, neuroblastoma, colorectal adenocarcinoma, and lung adenocarcinoma tissues, respectively. A scrutiny of the publicly accessible transcriptomic data obtained from similar cancer cell lines further confirmed the presence of the extra exon between exons 10 and 11 (Figure S1b, Supporting Information), which was not readily detected in non‐cancerous mammary or aortic epithelial cells and HeLa cells.

To better probe STIM1β expression in cancer tissues, we set out to develop an antibody specifically targeting the PAD region of STIM1β that was absent in normal STIM1. We found that the home‐made anti‐STIM1β polyclonal antibody against the epitope “AGSDDQSL” in the PAD domain exhibited excellent sensitivity and specificity toward STIM1β (Figure S2, Supporting Information). Consistent with the mRNA expression results (Figure 1d), we detected strong endogenous STIM1β immunostaining signals in U87 MG cells, but barely any in HEK293 and HeLa cells (Figure 1e). To further confirm STIM1β expression at the protein level in glioblastoma cells, we used magnetic beads conjugated with the anti‐STIM1β antibody to enrich endogenous STIM1*β* from the U87 cell lysate and indeed detected distinct peaks corresponding to the PAD fragment from STIM1β using mass spectrometry (Figure 1f), providing compelling evidence to support the physical presence of STIM1β in cancer cells. In parallel, we performed immunohistochemistry (IHC) staining for STIM1β in a paraffin embedded tissue array that contains 80 patient samples covering both normal and cancer tissues (Figure 1g and Figure S3, Supporting Information). We found that STIM1β was significantly upregulated in most cancer tissues compared to their corresponding normal tissues of origin, especially in glioblastoma and neuroblastoma (Figure S3, Supporting Information). These findings reinforce the conclusion that STIM1β expression is upregulated in cancers that originated from the brain, kidney, and reproductive organs. Compared to the relatively faint staining in normal or non‐cancer adjacent (NAT) brain tissues, STIM1β staining in glioblastoma isolated from different brain regions (*n* = 80) showed an over threefold increase in the average intensity (Figure 1h). Collectively, we have validated STIM1β overexpression in selected cancer cells and tissues both at the transcription and protein levels. Notably, the low expression of STIM1β in T and B cells makes it a more suitable anti‐cancer target than STIM1 to avoid potential side effects on the immune system.

### 2.2 STIM1β Contributes to SOCE and Is Prone to Be Activated

**Figure 2:**
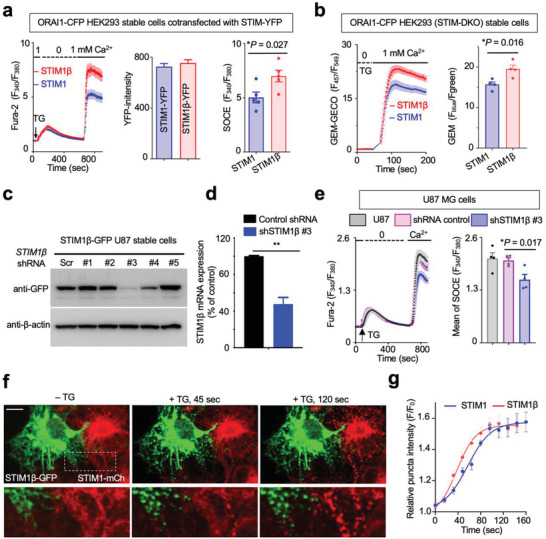
STIM1β contributes to SOCE and is prone to be activated. Data were shown as mean ± sem. Unpaired Student's *t*‐test. a) Ca^2+^ influx as monitored by Fura‐2 fluorescence in ORAI1‐CFP HEK293 stable cells cotransfected with STIM1‐YFP and STIM1β‐YFP, respectively, at comparable levels. Store depletion was induced by 1 µm thapsigargin (TG). Shown were representative traces (left), *n*  =  50 cells. The expression of STIM was at comparable levels based on YFP fluorescence intensities shown in the middle panel. The level of SOCE (*n* = 5) was summarized in the bar graph from five independent experiments (right), where each dot represents the average value from 30–60 cells. *p = 0.027. b) Comparison of Ca^2+^ entry in HEK293 STIM1/STIM2 double knockout (STIM‐DKO) cells expressing STIM1‐YFP or STIM1β‐YFP. The ratiometric Ca^2+^ sensor, GEM‐GECO, was used to monitor cytosolic Ca^2+^. *n* = 4 replicates, Each replicate counts for 30–60 cells. *p = 0.016. c) Immunoblot analysis to confirm the knockdown efficiency of STIM1β‐targeting shRNAs in STIM1β‐GFP U87 stable cells. *β*‐actin was used as loading control. d) Relative mRNA expression of STIM1β in U87 cells following shRNA‐mediated knockdown. *n* = 3. **p < 0.01. e) Representative recordings of Ca^2+^ influx as indicated by Fura‐2 in U87 cells upon shRNA treatment (left, the average value from ≈30–60 cells). Quantitation of SOCE for U87 cells was shown on the right. (*n* = 4; *p = 0.017 compared to control). f,g) Comparison of the activation kinetics of STIM1 and STIM1β. Representative confocal images (f) of mixed COS‐7 cells stably expressing STIM1‐mCh and STIM1β‐GFP, respectively, which showed protein clustering after store depletion triggered by 1 µm TG. g) Time course of STIM clustering. The half‐lives of activation were determined to be: STIM1β, *t*_1/2_ = 37.9 ± 6.7 s; STIM1, *t*_1/2_ = 56.9 ± 6.6 s. *n* = 12 cells from three independent experiments.

To explore the role of STIM1β in SOCE, we first monitored Ca^2+^ influx triggered by thapsigargin (TG) in ORAI1‐CFP HEK293 stable cells with STIM1‐YFP or STIM1β‐YFP transiently expressed at comparable levels, respectively (**Figure** **2**a). Given the extremely low or negligible mRNA expression of STIM1β in HEK293 cells, we reasoned this cell line could be used as a convenient model system with a STIM1β null‐like background. Overexpression of STIM1β neither induced a constitutive Ca^2+^ influx nor altered the Ca^2+^ release from ER Ca^2+^ stores (Figure S4, Supporting Information). We found that STIM1β elicited a stronger SOCE response than STIM1 did following TG‐induced passive store depletion in normal HEK293 cells (Figure 2a). To completely exclude the potential interference from endogenous STIM1 and STIM2, SOCE was also evaluated in HEK293 ORAI1 cells depleted of STIM1/2 (STIM‐DKO) with re‐expression of comparable levels of STIM1 or STIM1β. Again, we observed a more robust TG‐evoked Ca^2+^ influx in cells expressing STIM1β using GEM‐GECO (Figure 2b) or Fluo‐4 as Ca^2+^ indicators (Figure S5, Supporting Information). To exclude the possibility that the fused fluorescent protein tag might affect the activity of STIM1 variants, we further used a bicistronic mCh‐IRES (internal ribosomal entry site) vector to drive the co‐expression of mCherry (as expression marker) and untagged STIM1 or STIM1β. Again, we found that STIM1β induced larger SOCE in ORAI1‐CFP HEK293 STIM‐DKO cells (Figure S6a–c, Supporting Information). We subsequently repeated the similar experiments by using butylhydroquinone (BHQ, IC_50_: 5 µm), a weaker inhibitor of SERCA than TG (IC_50_: 10 nm), to trigger ER store depletion.^[^ ^16^ ^]^ A similar trend was noted, with STIM1β accounting for a stronger SOCE response (Figure S6d–f, Supporting Information). Next, we set out to examine the effect of STIM1β depletion on SOCE in human U87 glioblastoma cells bearing higher endogenous STIM1β (Figure 2c–e). shRNA‐mediated STIM1β knockdown was confirmed by both immunoblotting and qRT‐PCR (Figure 2c,d). In U87 cells treated with the most effective shRNA, we observed a 30% reduction in SOCE (Figure 2e). As a negative control, a scrambled shRNA expressed in the same cells did not affect the overall SOCE response. In aggregate, results from both overexpression and knockdown studies converge to support STIM1β as an important contributor to SOCE that outperforms STIM1 when expressed at comparable levels under the same stimulation conditions.

We further examined the behavior of STIM1β under physiological conditions by visualizing its subcellular distribution and monitoring puncta formation in real time. We expressed fluorescent protein (FP)‐tagged STIM1β in four distinct cell types (HEK293, HeLa, COS‐7, and U87) with varying amounts of endogenous STIM1β (Figure S7, Supporting Information). We found that, in the majority of transfected cells, STIM1β exhibited a smooth ER‐like tubular distribution, suggesting that STIM1β largely adopts an inactive conformation at rest, whereas acute TG treatment induced rapid punctate formation. Interestingly, constitutive puncta formation was observed in most HEK293 cells with ORAI1 overexpression (Figure S7b, Supporting Information), suggesting preactivation of STIM1β in the presence of excessive amount of ORAI1. Next, we compared the kinetics of puncta formation between STIM1 and STIM1β in mixed COS‐7 cell lines with a stable expression of comparable STIM1‐mCh and STIM1β‐GFP (Figure 2f). Time‐lapse imaging studies revealed that, compared to STIM1, STIM1β was able to form puncta more rapidly, with a half‐life of activation determined to be 37.9 ± 6.7 s (in contrast to 56.9 ± 6.6 s for STIM1; Figure 2f,g). Taken together, we have established STIM1β as a splice variant of STIM1 capable of more effectively eliciting SOCE and more prone to be activated following stimulation.

### 2.3 STIM1β‐CT Adopts a Partially Active State to Effectively Engage ORAI1

**Figure 3:**
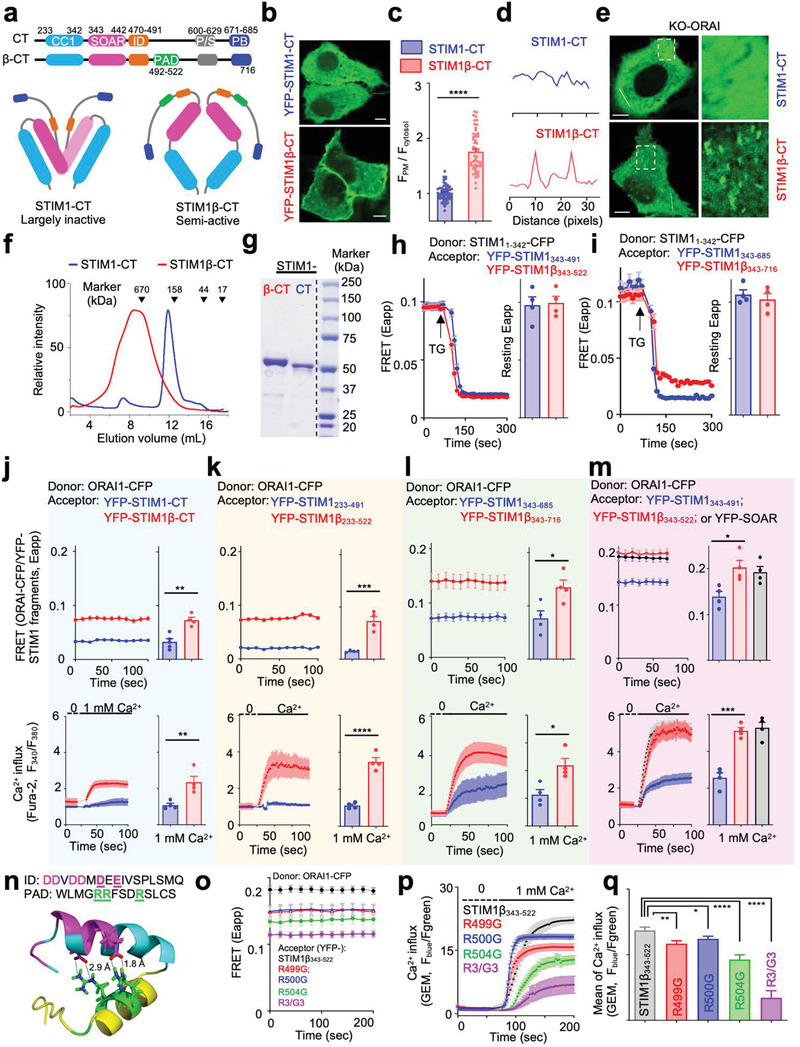
The cytoplasmic domain of STIM1β (STIM1β‐CT) is a more potent activator of ORAI1. a) Schematic representation of the domain architectures of STIM1‐CT and STIM1β‐CT. b,c) Confocal images (b) showing the subcellular distribution of YFP‐tagged STIM1‐CT and STIM1β‐CT. c) Quantification of the subcellular distribution ratio (PM/cytosol) of STIM1‐CT and STIM1β‐CT. *n*  =  54 cells from three independent experiments. d,e) YFP‐STIM1β‐CT displaying comet‐like patterns due to tracking of microtubule plus ends in ORAI‐KO HeLa cells. d) The fluorescence intensities (YFP) across the dashed line in panel (e) were plotted to evaluate the degree of comet formation. e) Selected confocal images showing different cytosolic distribution of STIM1‐CT and STIM1β‐CT. The selected regions (dashed boxes) were enlarged to aid visualization (right). Scale bar 5 µm. f,g) Elution profiles of purified recombinant STIM1‐CT and STIM1β‐CT when subjected to size exclusion chromatography. The molecular weights of standard protein samples were indicated above the elution profiles. g) The eluted fractions were collected and resolved on SDS‐PAGE. The isolated fractions (STIM1β‐CT at the elution volume of 7.5–8.5 mL; STIM1‐CT at 11.5–12.5 mL) were resolved by SDS‐PAGE. A dotted line indicated the separation between the sample lanes and the marker lane. h,i) FRET signals monitored in HEK293 cells co‐expressing STIM1_1‐342_‐CFP (donor) and YFP‐tagged STIM‐CT fragments (acceptor) before and after TG‐induced store depletion. STIM1_343‐491 _versus STIM1β_343‐522_ (h) and STIM1_343‐685 _versus STIM1β_343‐716_ (i). The resting FRET signals were plotted on the right, *n* = 4 independent experiments with 30–50 cells per experiment. j–m) Real‐time FRET signals (top) and constitutive Ca^2+^ entry (bottom) visualized in ORAI1‐CFP HEK293 stable cells expressing YFP‐tagged STIM‐CT variants, STIM1_233‐685 _versus STIM1β_233‐716_ (j), STIM1_233‐491 _versus STIM1β_233‐522_ (k), STIM1_343‐685 _versus STIM1β_343‐716_ (l), or STIM1_343‐491 _versus STIM1β_343‐522_ (m). The FRET values and Ca^2+^ responses (*n* = 4) were plotted on the right. Each dot represents the average value from 30–50 cells. n) The putative non‐covalent interactions between Asp/Glu negatively‐charged clusters of the ID domain and the positive Arg‐rich sequence within PAD in the model structure of the STIM1β_461‐523_. The distance between the side chains (N‐H—O) was 2.8–2.9 Å. o) Real‐time FRET signals in ORAI1‐CFP HEK293 stable cells expressing YFP‐STIM1β_343‐522_ (WT and the indicated mutants). *n* = 60–80 cells from three independent experiments. p,q) Constitutive Ca^2+^ entry was triggered by transition from 0 to 1 mm extracellular Ca^2+^ in ORAI1‐CFP HEK293 stable cells expressing YFP‐STIM1β_343‐522_ (WT and its mutants). *n* = 60–80 cells from three independent experiments. All error bars denote S.E.M. *p ˂ 0.05, **p ˂ 0.01, ***p ˂ 0.001, ****p ˂ 0.0001. Unpaired Student's *t*‐test.

To examine how the extra exon‐encoded domain (PAD) enhances the ability of STIM1β to form puncta and to induce stronger SOCE, we expressed the cytoplasmic domain of STIM1 (STIM1‐CT, amino acids 233–685) or STIM1β (STIM1β‐CT, amino acids 233–716) in HeLa cells (**Figure** **3**a). In contrast to the smooth cytosolic distribution of YFP‐STIM1‐CT, YFP tagged STIM1β‐CT prominently decorated the PM (Figure 3b,c). To verify whether the PM decoration of STIM1β‐CT depended on ORAI1, we examined the subcellular distribution of STIM1β‐CT in ORAI1/ORAI2/ORAI3 triple‐knockout HeLa cells (ORAI‐KO). We noted that STIM1β‐CT, similar to STIM1‐CT, failed to show a PM‐like distribution but mostly resided in the cytosol, suggesting the requirement of ORAI1 for PM targeting (Figure 3d,e). Because both STIM1 and STIM1β contain the EB1‐binding “TRIP” sequence and the degree of microtubule plus ending (+TIP) tracking capability is correlated with the oligomeric state of EB1‐binders,^[^ ^17^ ^]^ we compared the +TIP tracking behaviors of STIM1‐CT and STIM1β‐CT (Figure S8, Supporting Information). As anticipated, STIM1β‐CT displayed a striking comet‐like distribution pattern, a feature seen in the full‐length STIM1 due to constant microtubule plus‐end tracking^[^ ^18^ ^]^ but absent in STIM1‐CT due to limited self‐oligomerization (Figure S8 and Movie S1, Supporting Information). We and others have shown previously that STIM1‐CT adopts an inactive status with a “folded‐back” configuration via intramolecular trapping mediated by the coiled‐coil 1 (CC1) and STIM ORAI‐activating region (SOAR), thus precluding its self‐oligomerization with subsequent translocation toward the PM to engage ORAI1.^[^ ^1^, ^2^, ^19^ ^]^ Oligomerization is essential for switching STIM1‐CT from an inactive state to an active configuration,^[^ ^19^, ^20^ ^]^ a molecular step that has been reconstructed by us using an optogenetic engineering approach and taking advantage of the light‐induced oligomerization of the cryptochrome 2 (CRY2) photosensory protein.^[^ ^21^ ^]^ We therefore compared the behavior of CRY2‐STIM1‐CT and CRY2‐STIM1β‐CT before and after light stimulation. In HeLa cells co‐expressing GCaMP6s as a readout for Ca^2+^ signals, we found that both groups exhibited light‐triggered Ca^2+^ influx, but the STIM1β‐CT group showed a higher basal fluorescent intensity in the dark (Figure S9a, Supporting Information), implying partial pre‐activation of STIM1β‐CT. Furthermore, CRY2‐STIM1β‐CT was able to photo‐trigger Ca^2+^ influx with faster activation kinetics than CRY2‐STIM1‐CT (*t*_1/2, on_  =  28.9 versus 38.9 s; Figure S9b,c, Supporting Information). To further estimate the oligomeric state of STIM1β‐CT in vitro, we examined the behavior of both recombinant STIM1‐CT and STIM1β‐CT proteins by size‐exclusion chromatography (Figure 3f,g). STIM1β‐CT was found to be eluted much earlier than STIM1‐CT, implying its existence as a larger oligomer than the dimeric STIM1‐CT.^[^ ^20c^ ^]^ Taken together, these data corroborate the conclusion that STIM1β‐CT probably exists as a high‐order oligomer and adopts a partially activated conformation to enable a more rapid and potent activation of Ca^2+^ influx.

### 2.4 STIM1β‐CT Is a Potent Activator of ORAI1

The next question we asked is how the additional 31 residues of the PAD region perturbed the activity of STIM1‐CT. We first sought to determine whether PAD would affect the CC1‐SOAR interaction, which is known to mediate the intramolecular trapping of STIM1‐CT to prevent the exposure of SOAR for further oligomerization and subsequent association with ORAI1.^[^ ^1^, ^2^ ^]^ By utilizing a two‐component FRET assay developed by us to monitor CC1‐SOAR interactions in trans in live cells,^[^ ^19^, ^22^ ^]^ we found no significant difference in the basal FRET signals in cells co‐expressing the donors and acceptors (STIM1_1‐342_‐CFP as donor; STIM1_343‐491 _versus STIM1β_343‐522;_ or STIM1_343‐685 _versus STIM1β_343‐716_ as two sets of acceptors; Figure 3h,i and Figure S10a–d, Supporting Information). These findings suggest that the inclusion of PAD per se does not seem to affect the binding between ER anchored CC1 and isolated SOAR‐containing fragments. However, the whole STIM1β‐CT (STIM1β_233‐716_) showed bimodal distribution on ER and PM at rest (Figure S10e,f, Supporting Information) in HeLa cells coexpressing STIM1_1‐342_‐CFP, which suggests that STIM1β‐CT at least partially overcomes intramolecular autoinhibition and thus enables its SOAR domain to further interact with ER‐localized CC1 (STIM1_1‐342_) and PM localized ORAI in trans. Similar phenomena were also visualized on an activating mutant, STIM1‐CT (L258G), due to the introduced mutation abolishing CC1 structure and liberating SOAR domain.^[^ ^21^ ^]^ Thus, these results indicate that the PAD insertion may perturb CC1‐SOAR intramolecular interaction in the context of STIM1β‐CT.

We next assessed the role of PAD insertion in regulating the SOAR‐ORAI1 interaction by taking a subdomain approach. We monitored FRET and Ca^2+^ entry in ORAI1‐CFP HEK293 cells co‐expressing a series of YFP‐tagged STIM1‐CT subdomains (Figure 3j–m). For the constructs containing the autoinhibitory CC1 regions (STIM1_233‐686_ and STIM1_233‐491_ or the equivalent STIM1β versions; Figure 3j,k)_,_ both showed low FRET values with ORAI1‐CFP and exhibited negligible Ca^2+^ influx when switching the extracellular Ca^2+^ concentration from 0 to 1 mm without store depletion. Inclusion of PAD into these constructs enhanced the FRET signals and caused constitutive Ca^2+^ influx (Figure 3j,k), indicating that PAD insertion might destabilize the resting state of STIM1‐CT to cause partial pre‐activation. In addition, expression of YFP‐STIM1β_233‐522_ was found to elicit constitutive CRAC channel currents (*I*_CRAC_) in the presence of extracellular Ca^2+^ (Figure S11, Supporting Information), again confirming the partially activated state of STIM1β‐CT. For constructs without the CC1 domain (STIM1_343‐685_ and STIM1_343‐491_ or the equivalent STIM1β versions), all exhibited relatively high FRET signals and varying degrees of constitutive Ca^2+^ influx (Figure 3l,m). Notably, the construct STIM1β_343‐522_ showed the highest potency by matching the performance of SOAR, the minimal ORAI‐activating domain in STIM1. In comparison, the fragment STIM1_343‐491_, which encompasses SOAR and a downstream inhibitory domain (ID, STIM1_470‐491_), showed less FRET and spontaneous Ca^2+^ influx, suggesting a weaker engagement and activation of ORAI channels. The ID region is an inhibitory element other than the CC1 with a conserved negatively charged cluster that might trap STIM1 in a less active conformation. For instance, compared to SOAR/CAD (339‐444) alone, the ORAI‐activating ability of STIM1‐CT fragments containing the ID region (339‐475) was reduced by 50%,^[^ ^23^ ^]^ suggesting that the ID region can effectively inhibit SOAR's ability to engage ORAI1 and activate Ca^2+^ influx.^[^ ^23^ ^]^ Deletion of ID results in the liberation of SOAR and constitutive activation of Ca^2+^ influx.^[^ ^24^ ^]^ In the context of STIM1_233‐485_, the removal of ID has been shown to enhance the CRAC channel current from ≈4 to ≈13–15 pA pF^−1^.^[^ ^23^ ^]^ These findings prompted us to hypothesize that the insertion of PAD, although it does not disrupt the CC1‐SOAR interaction as described above (Figure 3h,i), might perturb the ID region to sabotage the co‐inhibitory machinery of STIM1‐CT. Indeed, a model structure of STIM1β_461‐525_ suggested potential non‐covalent interactions, such as salt bridges and hydrogen bonds between the ID and PAD regions (Figure 3n), which involved the negatively charged cluster in the ID (_475_ DDVDDMDEE_483_) and an Arg‐rich fragment within the PAD (_499_ RRFSDR_504_). We reasoned that the non‐covalent interactions between the ID and PAD motifs might account for the partial pre‐activation of STIM1β. To test this hypothesis, we subsequently created four neutralizing mutants (R499G, R500G, R504G, and R499G/R500G/R504G abbreviated as R3/G3) to disrupt these putative non‐covalent interactions in the context of STIM1β_343‐522_, anticipating to observe a stronger autoinhibitory phenotype, reflected in a reduction in the FRET with ORAI1, and lower Ca^2+^ entry. Indeed, compared with the wild‐type version, both single mutant (R499G, R500G, or R504G) and the triple mutant (R3/G3) led to a significant decrease in the FRET signals with ORAI1‐CFP and a 10–60% reduction in the ability to elicit a constitutive Ca^2+^ influx (Figure 3o–q), with the triple mutant exhibiting the strongest effects. When introduced into the full‐length STIM1β, the triple mutations (R3/G3) led to a marked reduction in TG‐triggered SOCE (Figure S6b,c, Supporting Information). Congruently, by taking a subdomain approach coupled with site‐directed mutagenesis, we have unveiled putative non‐covalent interactions between PAD and ID as a potential molecular mechanism to weaken the autoinhibitory machinery in STIM1‐CT, which may explain the pro‐activating capability of PAD encoded by the extra exon.

### 2.5 STIM1β Promotes Glioblastoma Cell Growth both In Vitro and In Vivo

**Figure 4:**
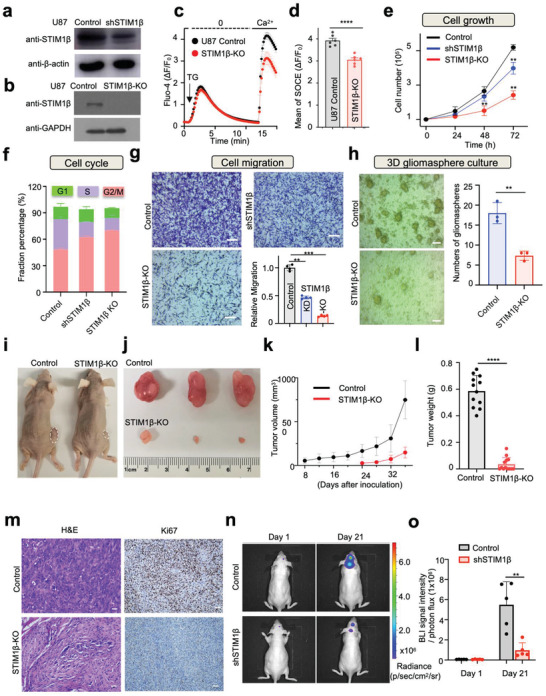
STIM1β depletion suppresses glioblastoma cell growth. Data were shown as mean ± sem. *p ˂ 0.05, ** *p* ˂ 0.01 ****p ˂ 0.0001. Unpaired Student's *t*‐test. a,b) Immunoblotting to confirm the knockdown (a) and knockout (b) of STIM1β in U87 cells. c,d) Evaluation of TG‐evoked Ca^2+^ influx in U87 cells (control and STIM1β knockout; (c)). d) Quantification of mean SOCE (*n* = 6 biological replicates). Approximately 30–60 cells were selected in each experiment. e–g) Effects of STIM1β knockdown (shSTIM1β) or knockout (STIM1β‐KO) on U87 cell growth (*n* = 3) (e), cell cycle (*n* = 2) (f), and migration revealed by the transwell assay (*n* = 4) (g). Scale bar, 50 µm. h) Effect of STIM1β knockout on the formation of gliomasphere in serum‐free neural stem cell culture media. Scale bar, 50 µm. i–l) STIM1β depletion inhibited U87 glioblastoma growth in a mouse xenograft model (*n* = 3). i) Representative images of mice bearing WT (control) or STIM1β KO U87 cell xenografts. j) Representative tumor images. k) Quantification of the tumor size at the indicated time points. l) Statistics of tumor weight. (*n* = 5–6 mice/group). m) HE and Ki‐67 staining of representative U87 xenograft tumors. Scale bar, 20 µm. n) Representative bioluminescence images of U87‐Luc and shSTIM1β U87‐Luc xenografts inoculated in the mouse brain. Nude mice were intracranially implanted with U87‐Luc and shSTIM1β U87‐Luc GBM cells (1 × 10^5^ cells/mouse). The tumor size was estimated by monitoring bioluminescence imaging (BLI). o) Quantification of BLI signals from the indicated groups (*n* = 5–6 mice/group).

The abnormal upregulation of STIM1β in glioblastoma and its pro‐activating property motivated us to further explore the potential pathological role of STIM1β in cancer. We first set out to examine the functional consequences of STIM1β knockdown (KD) or knockout (KO) in U87 cancer cells by taking shRNA mediated gene silencing or CRISPR/Cas9‐based gene disruption (KO) approaches. Both STIM1β KD and KO U87 cells (**Figure** **4**a,b) showed appreciable reduction (25–30%) in SOCE (Figures 2e and 4c,d). Using a colorimetric assay for cell proliferation, we found that STIM1β knockdown and knockout significantly suppressed the growth rate of U87 cells (Figure 4e). Cell cycle analysis by flow cytometry further revealed that STIM1β‐KD (shSTIM1β) and STIM1β‐KO U87 cells displayed a prominent cell cycle arrest at the G1 phase, with an accompanying decrease in the S phase (Figure 4f). Furthermore, both transwell migration and wound healing assays indicated that STIM1β depletion substantially impaired U87 cell migration (Figure 4g and Figure S12, Supporting Information). To more accurately mimic the clinical phenotype, we examined gliomasphere formation by resorting to 3D spheroid culture in serum‐free neural stem cell culture media.^[^ ^25^ ^]^ STIM1β knockout strongly inhibited the degree of gliomasphere formation (Figure 4h). Taken together, our results demonstrate that STIM1β knockdown or depletion inhibited the proliferation and migration of U87 cancer cells ex vivo.

To further validate the effects of STIM1β depletion on tumor growth in vivo, we subconsciously injected equal numbers of normal or STIM1β‐KO U87 glioblastoma cells into the dorsal flanks of nude mice and monitored tumor growth every 4 days for over 1 month. Tumor formation occurred much later in mice injected with STIM1β‐KO U87 cells compared to those inoculated with native U87 cells. In mice that formed tumors within the tested period, tumor weight and volume were substantially reduced in the STIM1β‐KO group compared to the control group with U87 xenografts (Figure 4i–l). H&E staining results revealed that xenograft tumors from the STIM1β‐KO group showed a less dense distribution of tumor cells. Consistent with our in vitro findings (Figure 4e), IHC staining of the proliferation marker Ki‐67 revealed substantially fewer proliferative cells in STIM1β‐KO U87 xenograft tumors compared to controls (Figure 4m). We further assessed the effects of STIM1β knockdown on tumor growth in a murine orthotopic GBM tumor model. Equal numbers of normal U87‐Luc or shSTIM1β U87‐Luc cells were implanted in the intracalvarium and monitored for growth by using bioluminescence imaging. Three weeks after implantation, the bioluminescent intensities of areas injected with shSTIM1β U87‐Luc cells were significantly lower than those arising from the control U87‐Luc cells (Figure 4n). Collectively, these in vivo findings reinforce the conclusion that STIM1β plays a tumor‐promoting role and STIM1β inactivation might provide an alternative approach to curtail tumor growth.

### 2.6 Discussion and Conclusions

Alternative splicing represents an economic mechanism to expand and diversify the function of signaling proteins.^[^ ^26^ ^]^ Meanwhile, emerging data suggest that aberrant splicing promotes tumor growth and thus can be exploited for the development of novel cancer biomarkers.^[^ ^27^ ^]^ In this study, we identified an alternatively spliced variant of STIM1, STIM1β, that is evolutionarily conserved among mammals but aberrantly upregulated in certain types of cancer, such as glioblastoma. The splicing event leads to the insertion of an extra exon encoding 31 residues to render STIM1 more prone to oligomerization/activation and able to engage and gate ORAI channels with higher efficacy. STIM1β is widely expressed across major human tissues, but its expression levels remain low when compared to conventional STIM1. Recently, Knapp et al. reported the same splice isoform of STIM1 that shows similar tissue distribution,^[^ ^28^ ^]^ with a higher expression ratio of STIM1β/STIM1 in the heart, testes, and kidney, and a much lower expression in immune cells. The extremely low expression of STIM1β in the immune system makes it a more ideal target for developing anti‐cancer therapeutics, as this promises to circumvent undesired immunosuppressive side effects associated with the unselective inhibition of aberrant ORAI‐STIM signaling in cancer cells.

**Figure 5:**
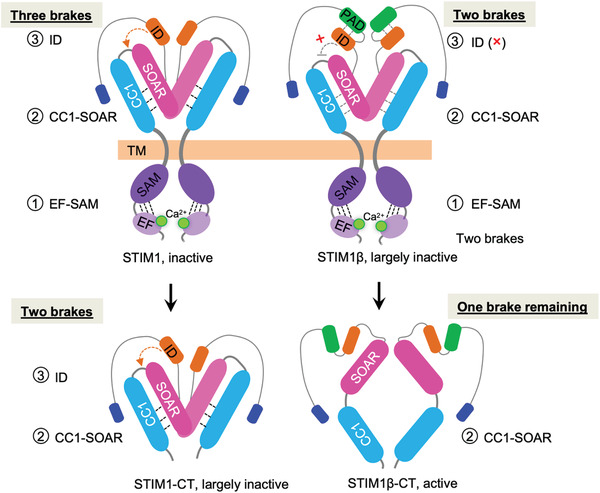
A tentative model to explain STIM1β activation. At the resting condition, the full length STIM1 contains all three inhibitory regions to prevent pre‐activation: 1) ER luminal EF‐SAM autoinhibition; 2) cytoplasmic CC1‐SOAR autoinhibition; and 3) inhibitory domain (ID), which work concertedly to force STIM1 adopting a folded‐back configuration and keep itself inactive. After removal of the luminal EF‐SAM domain, the STIM1 cytoplasmic domain (STIM1‐CT) remains largely inactive because of the existence of two remaining braking mechanisms. For STIM1β, the additional insertion of 31‐residue in the PAD region may perturb the inhibitory function of ID to compromise the autoinhibitory machinery in STIM1β. Although STIM1β still adopts a largely inactive status, its activation kinetics and gating ability to ORAI channels are greatly enhanced. Compared with the largely‐inactive STIM1‐CT, STIM1β‐CT assumes a conformation that is more prone for activation after removal/weakening of two inhibitory brakes. Please note that the oligomeric state, relative positioning and orientation of each domain in the cartoon are yet to be determined by further structural studies.

How does STIM1β modulate SOCE? Through a systematic dissection of the major steps during SOCE activation, we posit that the PAD region of STIM1β could potentially impact the co‐inhibitory ID domain of STIM1‐CT, thereby enabling STIM1β to more rapidly overcome autoinhibition after store depletion. The autoinhibitory machinery of STIM1 consists of at least three molecular “brakes”: the luminal EF‐SAM region (aa 63‒200),^[^ ^29^ ^]^ the coiled‐coil interaction between CC1 (aa 233‒342), and SOAR (aa 343‒442/448) in the juxta‐membrane cytosolic region,^[^ ^19^, ^30^ ^]^ and the ID region (aa 470‒491) downstream of SOAR(**Figure** **5**).^[^ ^24^ ^]^ Since the full‐length STIM1β still contains two brakes after perturbation of the ID brake imposed by PAD, it primarily adopts an inactive conformation. Nonetheless, in the presence of excessive amounts of ORAI1 to engage SOAR, the equilibrium tends to shift STIM1β toward an activated state, as reflected by the spontaneous formation of STIM1β puncta in ORAI1‐overexpressing cells (Figure S7b, Supporting Information). By comparison, STIM1β‐CT lacks both the luminal and ID brakes, thereby adopting a partially‐active configuration, as reflected by its constitutive association with PM when expressed in mammalian cells with endogenous levels of ORAI1. STIM1‐CT has been well characterized as a stable dimer in solution^[^ ^20c^ ^]^ and remains smoothly distributed in the cytosol (Figure 3). The PAD insertion converts dimeric STIM1‐CT to a higher‐order oligomer, as indicated by the elution profile in size‐exclusion chromatography in vitro and the notable appearance of comet‐like patterns due to microtubule plus‐end tracking in living cells. Other than STIM1β, another alternatively spliced variant of STIM1, STIM1L, contains the insertion of an actin‐binding domain (aa 515–620) downstream of the SOAR‐ID region.^[^ ^3b^ ^]^ STIM1L shows faster activation after store depletion due to forming permanent clusters with actin.^[^ ^3b^ ^]^ STIM1β exploits a different mechanism to rapidly switch into an active conformation. Our results indicate that the PAD insertion weakens the autoinhibition mediated by CC1‐SOAR in STIM1β‐CT (Figure S7f, Supporting Information), while sabotaging the co‐inhibitory ID domain to liberate SOAR domain to engage ORAI1 (Figure 3j–m). The structural determinants have been further narrowed down to potential electrostatic interactions and hydrogen bonds between a negative‐charged region within ID (_475_ DDVDDMDEE_483_) and a polybasic motif within PAD (_499_ RRFSDR_504_). Weakening such interactions via neutralization of positive charges in PAD (R499G/R500G/R504G) significantly reduces the pro‐activation capability of PAD (Figure 3n–q and Figure S6b,c, Supporting Information). Knapp et al. also claimed that the charged motif (_499_ RRFSDR_504_) is important for its differential activity toward the ORAI channels. While we focused more on the early activation steps of STIM1β per se, they examined the effects of this charged motif on STIM‐ORAI coupling with mutagenesis studies. They proposed that the amino acid D503 in this charged motif may interact with conserved residues (W76, R77, and K78) in the extended ORAI1 pore helix, and thus interfere with ORAI gating.^[^ ^28^ ^]^

Functionally, we have unveiled a splicing variant of STIM1 that shows a relatively high expression in certain types of cancer cells. The knockdown or deletion of STIM1β in U87 cells inhibited cell proliferation, cell cycle progression, migration, and tumor growth by arresting the cell cycle at the G1 phase, which prolongs the length of the cell cycle and decreases cell cycle reentry.^[^ ^31^ ^]^ Interestingly, the silencing of STIM1 by RNAi in U251 glioblastoma cells has been shown to induce G0/G1 phase cell cycle arrest and inhibit tumorigenicity in nude mice.^[^ ^14^ ^]^ STIM1 or ORAI1 deficiency has been reported to inhibit the proliferation and migration in several cancer types. For example, the knockout of STIM1 inhibits the proliferation (50–60%), colony formation (≈80%), and invasion (≈50%) of hepatocellular cancer SMMC7721 and HepG2 cells.^[^ ^32^ ^]^ STIM1 knockdown inhibits cell proliferation (≈40%) at day 3 post‐transfection into cervical cancer SiHa cells.^[^ ^33^ ^]^ In U87 MG cells, we found that knockout of both STIM1 and STIM1β variants (pan‐STIM1‐KO) caused ≈80% reduction in SOCE (Figure S13b,c, Supporting Information), as opposed to ≈20% reduction upon STIM1β depletion. Pan‐STIM1 knockout also significantly suppressed the growth of U87 cells and cell migration. The proliferation rate was slightly lower than that of STIM1β‐KO U87 cells, but no statistical significance was detected between pan‐STIM1‐KO and STIM1β‐KO groups (Figure S13c, Supporting Information). In summary, STIM1β depletion inhibited SOCE, cell proliferation and migration by ≈20%, ≈70%, and ≈45%, while the suppressive effects imposed by pan‐STIM1 depletion were ≈80%, 78%, and 82%, respectively (Figure S13, Supporting Information). These findings imply that conventional STIM1‐mediated Ca^2+^ signaling is more critical for cell migration, but STIM1β alone seems to play a major role in regulating glioma cell growth.

Notably, genetic depletion of STIM1β in U87 cells caused 25–30% reduction in SOCE (Figures 2e and 4c,d) but led to a more pronounced inhibition of tumor growth in U87 xenografts (Figure 4i–o). A similar phenomenon was observed in PC‐3 prostate cancer cells with abundant ORAI3 expression.^[^ ^34^ ^]^ Even though ORAI3 depletion in PC‐3 cells caused a minor reduction in SOCE, it significantly reduced tumor growth. A plausible explanation is that alterations of ORAI1/ORAI3 ratio,^[^ ^34^ ^]^ or changes in expression ratios of ORAI/STIM variants,^[^ ^7^ ^]^ may disrupt the dynamic equilibrium of SOC channels that function as pro‐oncogenic switches in certain types of cancer.^[^ ^35^ ^]^ It is likely that augmented STIM1β expression could lead to a similar scenario via heterotypic interactions with other STIM/ORAI proteins. Alternatively, it is also likely that other unknown binders of STIM1β might further regulate tumor growth. Finally, given that previously used siRNA or other knockdown/knockout strategies will unbiasedly target both normal STIM1 and STIM1β, it remains imperative to revisit some earlier studies to sort out the contribution of each splice variant to cancer and other pathological conditions associated with augmented STIM1 signaling.

## 3 Experimental Section

### Ethics Statement

The studies using human tissues were approved by the Ethical Review Board of Sir Run Run Shaw Hospital, College of Medicine, Zhejiang University. The used human cell lines were abided with institutional guidelines on human cell research and the approved protocol by the Institutional Review Board. Animal experiments were approved by the Institutional Animal Care and Use Committee (IACUC) at the Department of Medical Oncology, Laboratory of Cancer Biology, Institute of Clinical Science, Sir Run Run Shaw Hospital, College of Medicine, Zhejiang University (ZJU2015‐220‐01).

### Cells, Antibodies, and Reagents

HeLa (CRM‐CCL‐2), HEK293 (CRL‐1573), COS‐7 (CRL‐1651), U87‐MG (HTB‐14), SK‐N‐SH (HTB‐11), Caco‐2 (HTB‐37), Astrocyte (CRL‐2541), and A549 (CCL‐185) cells were purchased from American Type Culture Collection (ATCC, Manassas, VA). U87 cell line that stably expressing luciferase (U87‐Luc) was purchased from Shanghai SunBio Biomedical technology CO., LTD. All cells were maintained in DMEM or recommended medium with 10% fetal bovine serum (FBS) according to culture instructions provided by ATCC. STIM1/STIM2 double knockout (STIM‐DKO) HEK293 cells and ORAI knockout HeLa cells (ORAI‐KO) were prepared by using the CRISPR/Cas9 genome editing technology with sgRNA inserted into the lentiCRISPRv2 vector (Addgene#52961).^[^ ^36^ ^]^ Antibodies used in western blot immunostaining include primary antibodies against GFP or YFP (#sc‐8334) and *β*‐actin (#sc‐47778), purchased from Santa Cruz Biotechnology (Dallas, TX). Anti‐GAPDH (#PA1‐16777) was purchased from Thermo Fisher Scientific (Waltham, MA). Mouse monoclonal STIM1 antibody and anti‐STIM1 antibody (A‐8, #sc‐166840, against STIM1_441‐620_) were purchased from Santa Cruz Biotechnology (Dallas, TX, USA). Another anti‐STIM1 antibody (#610954) recognizing the N‐terminal fragment STIM1_25‐195_ was obtained from BD Biosciences (USA). The polyclonal antibody specifically recognizing STIM1β (epitope: AGSDDQSL) was developed from immunization in rabbits (NeoScientific, Cambridge, MA, USA). The transfection reagent Lipofectamine 3000 was purchased from Life Technologies (Carlsbad, CA, USA). Isopropyl‐*β*‐D‐thiogalactopyranoside (IPTG), thapsigargin (TG), Di‐tert butylhydroquinone (BHQ), and other cell culture reagents were purchased from Sigma‐Aldrich (St. Louis, MO, USA). The Gel Filtration Standard was purchased from Bio‐Rad (Hercules, CA, USA).

### Molecular Cloning and Plasmid Construction

Full‐length human STIM1β was amplified from a cDNA library synthesized from RNA isolated from U87 MG cells. Full‐length STIM1β and STIM1 were then subcloned into pEYFP/CFP/mCherry‐N1 vectors (Clontech; Mountain View, CA, US) at the Xhol and BamHI sites, respectively. KOD Hot Start DNA polymerase was used for PCR amplification and purchased from EMD Millipore (Burlington, MA, USA). Restriction enzymes and T4 DNA ligase kit were purchased from New England BioLabs (Ipswich, MA, USA). For GFP‐STIM1β, STIM1β was inserted into pEGFP‐C1. For untagged STIM1β expression using a bicistronic vector, the mCh‐IRES vector was obtained from Addgene (#75368), with STIM1 and STIM1β sequences inserted individually between the XhoI and XmaI sites. The various cytoplasmic fragments of human STIM1 and STIM1β were amplified by standard PCR and subsequently inserted into the pEYFP‐C1 vector at the Xhol and EcoRI sites or into pEYFP‐N1 using the Xhol and BamHI sites. ORAI1‐CFP and ORAI1‐mCh were made by inserting ORAI1 into pECFP/mCherry‐N1 vectors. STIM1_1‐342_‐CFP was prepared by standard PCR and ligated with pECFP‐N1 vector at the Xhol and BamHI sites. mCh‐ORAI1 was made by inserting mCherry between the BamHI and EcoRI restriction sites and human ORAI1 between the EcoRI and XhoI sites in the pCDNA3.1(+) vector (Life Technologies, Carlsbad, CA, USA). For CRY2 fused to the STIM1β cytoplasmic tail, the PCR fragments were amplified and used to replace STIM1_233–685_ in the previously prepared construct of mCh‐CRY2‐STIM1_233–685_ via restriction enzyme digestion.^[^ ^21^ ^]^ ORAI1‐CFP was made by inserting ORAI1 in the pECFP‐N1 vector between the Xhol and BamHI restriction sites. For recombinant protein expression constructs in E. coli, the sequence of STIM1_233‐685_ and STIM1β_233‐716_ were amplified via PCR and cloned into the pPro‐EX‐HTb vector (Life Technologies) between the BamHI and XhoI sites for expression as (His)_6_‐STIM1_233‐685_ and (His)_6_‐STIM1β_233‐716_ proteins. For mutations, the QuikChange Multi Site‐Directed Mutagenesis Kit was obtained from Agilent Technologies (Santa Clara, CA, USA) was used.

### PCR and qRT‐PCR Analysis

Total RNA of human normal tissue was purchased from Clontech (#636532, #636525, #636529, #636531, #636524, #636577, #636527, #636550, #636533, #636576, #636742, #636743; Mountain View, CA). Buffy coats of blood from healthy donors (from the Gulf Coast Regional Blood Center, Houston, TX) were used for isolation of human peripheral blood mononuclear cells (PBMCs) by density‐gradient centrifugation with Lymphoprep (Nycomed Pharma, Oslo, Norway). All blood samples were anonymized. The use of PBMCs was in accordance with institutional guidelines on human cell research and an approved protocol by the Institutional Review Board. Human T cells and B cells were isolated from PBMCs using Dynabeads Untouched Human T cells and B cells kit (ThermoFisher Scientific, Waltham, MA). Total RNA from human PMBCs, T and B cells was isolated using Trizol reagent. The first‐strand cDNA was generated from total RNA using oligo‐dT primers and SuperScript III Reverse Transcriptase (Thermo Fisher Scientific). PCR reactions were performed on a T100 PCR Thermal Cycler with specific primers and DreamTaq Green PCR Master Mix (Thermo Fisher Scientific). Real‐time qPCR was performed on the ABI Prism 7000 analyzer (Applied Biosystems, Foster City, CA) with the PowerUp SYBR Green Master Mix (Thermo Fisher Scientific). Target gene expression values were normalized to human GAPDH.

The primers used for qRT‐PCR are as follows:

hSTIM1_Fwd‐1: CAACCCTGCTCACTTCATCA
hSTIM1_ Rev‐1: GGCTAGGGGACTGCATGG
hSTIM1_Rev‐1β: (GCGGATGTAGAGCAGAGAGA)
hSTIM1 _Rev‐2: CTGGCGGTCACTCATGTG
GAPDH_Fwd: GCA CCG TCA AGG CTG AGAAC
GAPDH_Rev: TGG TGA AGA CGC CAGTGG A

### Confocal Imaging

Cell lines used for live cell imaging or immunostaining include HEK293, HeLa, COS‐7, and U87 cells. All cells were seeded on 35‐mm bottom‐glass growth dishes (Cellvis, Mountain View, CA, USA) in DMEM (Sigma) supplemented with 10 mm HEPES and 10% heat‐inactivated FBS. Transfections were performed with Lipofectamine 3000 (Life Technologies) following the manufacturer's protocol. To aid efficient and stable puncta formation, the DMEM medium was replaced by pre‐warmed Ca^2+^‐free Hank's balanced salt solution (HBSS) before imaging. 1 µm thapsigargin was used to trigger store depletion. Live cell imaging was performed at room temperature with a 60× oil or 40× oil lens on an inverted Nikon Eclipse Ti‐E microscope customized with A1R‐A1 confocal modules using argon‐ion (405 and 488 nm) and helium‐neon (543 nm) or diode (561 nm) as laser sources. Image analysis was performed using the Nikon, NIS‐element AR version 4.5 or Image J (NIH).

To compare the activation kinetics of STIM1 and STIM1β, the COS‐7 cells stably expressing STIM1‐mCh and STIM1β‐GFP were mixed. After 12 h, the views containing both STIM1 and STIM1β were selected to monitor with the footprint of cells. 1 µm thapsigargin was used to trigger store depletion and STIM1 activation. The kinetics of puncta formation was calculated by measuring the clustering intensity from Time‐Lapse images.

### Real‐Time Intracellular Ca^2+^ Measurements

For measurements of cytosolic Ca^2+^ signals using the ratiometric genetically encoded Ca^2+^ indicator GEM‐GECO^[^ ^37^ ^]^ or Fura‐2, cells grown on round coverslips were imaged with a ZEISS observer‐Z1 microscope equipped with X‐Cite 120‐Q (Lumen dynamics) light source, Semrock Bright Line filter sets CFP (438 ± 12 Ex/482 ± 16 Em), YFP (500 ± 12Ex/542 ± 13.5Em), FRETraw (438 ± 12Ex/542 ± 13.5Em), 40× oil objective (N.A. 1.3), and Axiocam 506 mono Camera (Zeiss). The imaging system was controlled by SlideBook 6 software (Intelligent Imaging Innovations (3i) Inc.).

Dye loading and imaging of Fura‐2 were as described earlier.^[^ ^36^ ^]^ GEM‐GECO signals excited by 387 ± 12 nm were collected at 542 ± 13.5 nm (F_549_) and 457 ± 25 nm (F_457_). A YFP‐to‐GEM_542_ bleed through factor of 0.4 was used to correct contamination of GEM signals by YFP fluorescence. The dye‐loading solution or imaging solution contained 107 mm NaCl, 7.2 mm KCl, 1.2 mm MgCl_2_, 1 mm CaCl_2_, 11.5 mm glucose, and 20 mm HEPES–NaOH, pH 7.4. To keep cells healthy, for WT or STIM1/2 double KO stably expressing ORAI‐CFP stable cells (Orai1 or SKO) transfected with constitutively active STIM1 constructs, up to 1.8 mm EGTA was added into the culture medium 1 h after transfection. Cells were kept in 0.3 mm Ca^2+^, nominally Ca^2+^‐free, or 300 µm EGTA imaging or loading solution before recording. All experiments were carried out at room temperature. 1 µm thapsigargin was used to induce store depletion. Traces shown are representative of at least three independent repeats with each including 30–60 single cells.

For measurements of cytosolic Ca^2+^ signals using Fluo‐4 AM dye (Thermo Fisher Scientific), ORAI1‐CFP HEK293 (STIM‐DKO) stable cells transiently expressed mCh‐IRES‐STIM variants or U87 cells with or without depletion of STIM1β were incubated with 5 µm Fluo‐4 for 25–30 min at 37 °C with dye‐loading solution containing 0.3 mm Ca^2+^. After washing, cells were incubated for a further 30 min to allow complete de‐esterification of intracellular AM esters. Fluorescence imaging was recorded on a Nikon Eclipse Ti‐E microscope.

### Fluorescence Resonance Energy Transfer (FRET) Measurements

FRET experiments were performed using the above‐mentioned ZEISS observer‐Z1 imaging system. Semrock Bright Line filter sets CFP (438 ± 12Ex/482 ± 16Em), YFP (500 ± 12Ex/542 ± 13.5Em), FRETraw (438 ± 12Ex/542 ± 13.5Em), were used to collect CFP, YFP, and raw FRET images (FCFP, FYFP, and Fraw, respectively) every 10 s with SlideBook 6.0 Software. The system calibration was described earlier.^[^ ^36^ ^]^ The system‐independent apparent FRET efficiency, *E*_app_, was calculated with MATLAB 2014a and plotted with GraphPad Prism 8 software.

### Patch Clamp Recording

Patch‐clamp recordings were performed on HEK ORAI1‐CFP cells transiently expressing YFP‐STIM1_233‐491_ or YFP‐STIM1β_233‐522_ as previously described.^[^ ^38^ ^]^ The pipette solution contained (millimolar): 135 Cs‐Aspartate, 10 HEPES, 8 MgCl_2_, 10 BAPTA (PH 7.2 with CsOH). The 10 mm Ca^2+^ bath solution contained (millimolar): 115 NaCl, 4.5 KCl, 5 HEPES, 10 Glucose, 10 TEA (tetraethylammonium chloride), and 10CaCl_2_ (PH 7.4 with NaOH). The Ca^2+^‐free bath solution contained (millimolar): 115 NaCl, 4.5 KCl, 5 HEPES, 10 Glucose, 10 TEA, and 10 MgCl_2_ (PH 7.4 with NaOH). Currents were recorded with standard whole cell configuration using the EPC‐10 amplifier controlled with PatchMaster software (HEKA). A +10 mV junction potential compensation was applied to correct the liquid junction potential. The membrane potential was held at 0 mV after establishments of whole‐cell configuration, and 50 ms voltage ramps from −100 to +100 mV were delivered every 2 s. At least 6 cells for each condition were collected. All data were exported using FitMaster, analyzed with Matlab 2014b, and plotted with GraphPad Prism 8.

### Immunofluorescence and Immunohistochemistry (IHC)

HEK293, HeLa, and U87 MG cells were grown on 35‐mm glass‐bottom dishes. After 24 h, cells were fixed in 4% paraformaldehyde (PFA) solution in PBS at room temperature for 15 min and then permeabilized at room temperature with 0.1% Triton X‐100/PBS solution, and subsequently blocked with 3% BSA for 60 min. For the overexpression of target proteins, cells were fixed 16 h after transfection. The dish was incubated with anti‐STIM*β* antibody diluted 1:200 (endogenous) or 1:1000 (overexpression) overnight at 4 °C. Cells were then washed three times with PBS and incubated with Donkey anti‐Goat Secondary Antibody, Alexa Fluor 488 or 568 with 1:1000 dilution for 1 h. The nucleus was stained with DAPI for 2 min. The cells were then captured using a Nikon Eclipse Ti‐E microscope and analyzed with the Nikon NIS‐Elements AR package software.

The STIM1β levels in brain tissue were evaluated by IHC using anti‐STIM1β antibody on a purchased human brain cancer tissue array (GL805a, US Biomax Inc.). The tissue array slide was deparaffinized and stained with an anti‐STIM1β antibody. IHC‐stained tissue images were obtained and evaluated at an inverted Nikon Eclipse Ti‐E microscope with 40× oil lens. The relative expression of STIM1β was normalized against the sample with the highest immunostaining intensity.

### Recombinant Protein Expression, Purification, and Characterization

*Escherichia coli* strain BL21 (DE3) cells (EMD Millipore) were transformed with (His)_6_‐STIM1_233‐685_ or (His)_6_‐STIM1β_233‐716_ respectively, and grown at 37 °C in LB medium with ampicillin, then induced by the addition of 0.5 mm IPTG when OD600 of the medium reached 0.6–0.8. The cells were incubated for another 12–16 h at 16 °C. The harvested cells were resuspended with buffer containing 20 mm Tris‐HCl pH 7.4, 10 mm imidazole, 200 mm NaCl, 2 mm TCEP, and sonicated. The supernatant was applied to Ni^2+^‐nitrilotriacetic acid (Ni‐NTA)‐agarose resin (Qiagen). The recombinant proteins were eluted in 20 mm Tris pH 7.4, 250 mm imidazole, 150 mm NaCl, 1 mm TCEP, and further purified by gel filtration on a Superose 6 10/300 GL column (GE Healthcare). The Gel Filtration Standard was used to indicate the size of purified proteins in solutions. The isolated fractions of STIM1‐CT and STIM1β‐CT were collected and further verified by SDS‐PAGE after Coomassie Brilliant Blue R‐250 staining.

### Western Blot and Mass Spectrometry Analysis

STIM1‐GFP and STIM1β‐GFP U87 stable cells or STIM1β knockout U87 cells were washed in cold PBS for 3 times and lysed with buffer including 20 mm Tris‐HCl (PH 7.4), 150 mm NaCl, 1 mm EDTA, 1 mm EGTA, 1% Triton X‐100, 2.5 mm sodium pyrophosphate, and 1 mm *β*‐glycerophosphate, supplemented with protease inhibitor cocktail and phosphatase inhibitor cocktail (Sigma), for 30 min at 4 °C. After centrifugation at 20 000x g at 4 °C for 15 min, equivalent sample amounts were separated by SDS‐PAGE, followed by transferring to PVDF membranes and probing with anti‐STIM1β and other indicated primary antibodies as well HRP‐conjugated secondary antibodies.

U87 cell lysate was enriched for STIM1β by incubation with the authors’ anti‐STIM1β antibody and with Goat Anti‐Rabbit IgG Magnetic Beads (New England Biolabs Inc.). Proteins concentrated on the beads were boiled, eluted, and analyzed by gel electrophoresis and Western blot. Then, the portion of the gel around the predicted STIM1β molecular weight was sliced and subjected to in‐gel digestion with trypsin. The prepared samples were further analyzed by liquid chromatography with tandem mass spectrometry (LC–MS/MS) using a Linear Ion Trap mass spectrometer (Thermo Fisher Scientific) equipped with a nano‐spray source and high‐performance liquid chromatography. A full‐scan survey mass spectrometry experiment was performed and the most abundant ions were further analyzed by MS‐MS scan events with a normalized collision energy of 35%. The obtained precursors and those of the fragment ions were entered into a database to facilitate further analysis.

### Stable Cell Line Generation

For STIM stable cells, the PCR products of STIM1β or STIM1 were digested with PmeI and BamHI, respectively, and cloned into the pWPXLd lentiviral vector with EGFP florescent tag (Addgene#12258). Then the florescent protein GFP was replaced by mCherry with BamHI and EcorI resulting STIM1β‐mCh or STIM1‐mCh in the pWPXL vector. The lentiviral particles were prepared by cotransfection of target plasmids with the packing and envelope plasmids psPAX2 (Addgene #12260) and pMD2.G (Addgene#12259) in HEK293T cells. After 48 h, the supernatants were harvested and applied to COS‐7 and U87 cells. For ORAI‐CFP stable cells with STIM1/2 double KO (STIM‐DKO), STIM‐DKO cells^[^ ^36^ ^]^ were transfected with ORAI1‐CFP, and selected with 100 µg mL^−1^ G418. Subsequently, single cell clones were prepared by selected cells using limiting dilution cloning in 96 well plates. The stable clones were further characterized by confocal imaging and western blotting.

### sgRNA‐Directed Knockout of STIM1β and STIM1 Using the CRISPR/Cas9 Genome Editing Tool

The guide RNA (5’‐GGACTGATCATCCGAGCCGG‐3’, negative strand) targeting the novel splice in STIM1β was designed with an online tool (http://crispr.mit.edu) and inserted into the BsmBI site of the LentiCRISPRv2GFP vector (Addgene# 82416) to generate LentiCRISPRv2GFP‐sgSTIM1β. The sgRNA‐containing plasmids with pMD2.G and psPAX2 package plasmids were co‐transfected into packaging HEK293T cells. 48 and 72 h after transfection, the viral supernatants were filtered through a 0.45 µm filter and used to infect U87 cells twice over two consecutive days, followed by fluorescence‐activated cell sorting (FACS)‐based cell sorting for GFP (+) cells. Cells were harvested and seeded on a 96‐well plate. Single colonies were selected and later expanded to a 24‐well plate. Genomic DNA from stable cell clones was extracted using the Quick‐gDNA kit (Promega) and the genomic region flanking the CRISPR/Cas9 targeting site for STIM1β was amplified via PCR. The PCR products were purified using the Gel DNA Recovery Kit (Promega) according to the manufacturer's instructions. Purified PCR products were sent for sequencing by Eurofins Genomics for further validation. The STIM1β‐KO cells were also verified by western blot with an anti‐STIM1β antibody. Pan‐STIM1 knockout was carried out using the similar procedures with the guide RNA sequence targeting the N‐terminal region shared by both STIM1 and STIM1β: 5’‐GTATGCGTCCGTCTTGCCCTG‐3’.

### Generation of shRNA Transduced U87 Cells

To generate the STIM1β stable knockdown U87 cell line, five pairs of shRNA against the STIM1β novel splice sequence were used to generate lentiviruses in HEK293T cells with pLKO.1‐mCherry (Addgene#128073) vector and packaging plasmids pMD2.G and psPAX2. For rapid screening of shRNA pairs, the viral supernatants were first used to infect STIM1β‐GFP stable U87 cells. The knockdown effect of shRNA pairs was verified by western blotting analysis and confocal imaging. The validated shRNA pair (STIM1β#3_shRNA) was further transduced in U87 cells. After two rounds of transfection with viral supernatants, U87 cells expressing mCherry were sorted with fluorescence‐activated cell sorting (FACS). STIM1β knockdown efficiency in U87 native cells was assessed by quantitative PCR. U87‐shSTIM1β‐Luc cells used for orthotopic transplantation were established similarly though transduction of U87‐Luc cells with STIM1β#3 shRNA.

The shRNA sequences were as follows:

Control_shRNA_Fwd: CCGG CCTAAGGTTAAGTCGCCCTCG CTCGAG CGAGGGCGACTTAACCTTAGG TTTTTG
Control_shRNA_Rev: AATTCAAAAA CCTAAGGTTAAGTCGCCCTCG CTCGAG CGAGGGCGACTTAACCTTAGG
STIM1β#1_shRNA_Fwd: CCGG TGCTGCCTGGCTGATGGGGCG CTCGAG CGCCCCATCAGCCAGGCAGCA TTTTTG
STIM1β #1_shRNA_Rev: AATTCAAAAA TGCTGCCTGGCTGATGGGGCG CTCGAG CGCCCCATCAGCCAGGCAGCA
STIM1β#2_shRNA_ Fwd: CCGG AGGTTCAGTGACCGCTCTCTC CTCGAG GAGAGAGCGGTCACTGAACCT TTTTTG
STIM1β#2_shRNA_Rev: AATTCAAAAA AGGTTCAGTGACCGCTCTCTC CTCGAG GAGAGAGCGGTCACTGAACCT
STIM1β#3_shRNA_ Fwd: CCGG ATCAGTCCCTCTGGAAATACC CTCGAG GGTATTTCCAGAGGGACTGAT TTTTTG
STIM1β#3_shRNA_Rev: AATTCAAAAA ATCAGTCCCTCTGGAAATACC CTCGAG GGTATTTCCAGAGGGACTGAT
STIM1β#4_shRNA_ Fwd: CCGG ATGATCAGTCCCTCTGGAAAT CTCGAG ATTTCCAGAGGGACTGATCAT TTTTTG
STIM1β#4_shRNA_Rev: AATTCAAAAA ATGATCAGTCCCTCTGGAAAT CTCGAG ATTTCCAGAGGGACTGATCAT
STIM1β#5_shRNA_ Fwd: CCGG AGTCCCTCTGGAAATACCCCG CTCGAG CGGGGTATTTCCAGAGGGACT TTTTTG
STIM1β#5_shRNA_Rev: AATTCAAAAA AGTCCCTCTGGAAATACCCCG CTCGAG CGGGGTATTTCCAGAGGGACT

### Wound Healing Assay

A total of 1 × 10^6^ U87 cells were seeded into 6‐well plates and allowed to attach overnight. A wound was made in the confluent cell layer by horizontally scratching the well using a sterile 200‐µL pipette tip. Cell migration was checked until the wound healed in one of the samples. Images of the scratch area were captured using a microscope (Nikon). The remaining wound area was measured using ImageJ software.

### Colorimetric Assay of Cell Proliferation

Cell proliferation was determined by using a conventional MTS assay. Cells were seeded in 12‐well plates at a density of 1 × 10^5^ cells per well. 50 µL MTS (Promega, Madison, USA) was added to each well at different time points. After a 2‐hour incubation, the culture medium was transferred to 96‐well plates (200 µL per well). The absorbance was measured in an ELX800 Micro Plate Reader (Bio‐Tek Instruments, USA) at 490 nm.

### Transwell Migration Assay

Transfected cells were adjusted to a density of 1 × 10^6^ mL^−1^ in a serum‐free DMEM medium. A total of 200 µL of cell suspension was seeded into the upper side of the transwell chamber (Corning, NY, USA). In the bottom part, 1 mL of the DMEM medium containing 20% FBS was added. After 24 h of incubation, non‐migrated cells were removed from the top of the chamber with a cotton swab. Cells at the bottom of chamber were fixed with methanol for 10 min, dyed with 0.1% crystal violet for 30 min. After two washes with PBS, migrated cells were photographed and counted in randomly selected fields.

### Flow Cytometric Analysis for Cell Cycle Distribution

WT, STIM1β‐KD, or STIM1β‐KO U87 cells (5 × 10^5^ cells/well) were collected, washed twice in cold PBS, then fixed in cold ethanol (70% w/w) at 4 °C overnight. The next day, the cells were washed with PBS and incubated with DAPI for 10 min. Finally, after washing with PBS, the percentage of cells in each phase of the cell cycle was detected by flow cytometry. The data was analyzed with FlowJo software.

### Gliomasphere Culture

WT or STIM1β‐KO U87 cells were seeded in 24‐well plates at a density of 2×10^4^ cells/well in a serum‐free neural stem cell culture media, containing DMEM/F12, B27 (1×, Gibco), recombinant human epidermal growth factor (rhEGF, 20 ng mL^−1^, Peprotech), basic fibroblast growth factor (bFGF, 20 ng mL^−1^), leukemia inhibitory factor (LIF, 10 ng mL^−1^), and cultured for 20 days. The culture medium was changed every 2–3 days and the gliomaspheres were photographed and counted in randomly selected fields.

### Xenograft Tumor Models

Animal experiments were approved by the Institutional Animal Care and Use Committee (IACUC) at Institute of Biosciences and Technology, College of Medicine, Texas A&M University, and institutional board of Department of Medical Oncology, Laboratory of Cancer Biology, Institute of Clinical Science, Sir Run Run Shaw Hospital, College of Medicine, Zhejiang University (ZJU2015‐220‐01). Briefly, 100 µL U87 or U87‐STIM1‐KO cells (at a density of 2 × 10^4^ µL^−1^) were subcutaneously injected into the posterior limbs of the nude mice. About a week later, a palpable lump could be seen in the group of mice injected with U87 cells. The tumor size was measured with calipers every three days and volume was calculated using the formula *V* = *LW* ^2^/2, where *L* is the largest diameter and *W* is the perpendicular diameter. When one tumor reached 1.5 cm in diameter, mice were euthanized and the tumors were harvested, weighed and subjected to paraffin embedding. The xenograft experiment was repeated for 3 independent times with 10–12 mice in each time. As no tumors formed in some of the mice injected with U87‐STIM1‐KO when sacrificed, the volume was counted as 0.

Xenograft tumor tissue was isolated from nude mice and then fixed with 10% PFA overnight at 4 °C. Then, the tumor tissue was embedded in paraffin using standard procedures as follows: 70% ethanol for 1 h, 80% ethanol for 1 h, 95% ethanol for 1 h, 100% ethanol for 1 h × 2, 50% ethanol + 50% xylene 15 min, xylene 15 min × 2, paraffin I 30 min, paraffin II 30 min, and paraffin III 30 min. 5 mm sections were cut, processed (deparaffinization, rehydration, antigen retrieval), and stained with hematoxylin and eosin (HE) dye or antibodies directed against the cell proliferation marker Ki67 (Abcam; Cat # ab8191).

To generate the orthotopic GBM model in the brain, 6‐week‐old male nude mice were anesthetized and poked a small hole with sharp needle at the position of 1 mm anterior to fontanelle and 2 mm to the right side with the stereotactic instrument. The micro syringe was vertically inserted into 3.5 mm, and retracted 0.5 mm after staying for 5 min. 5 µL (1 × 10^5^ cells/µL) U87‐Luc or U87‐shSTIM1β‐Luc cell suspensions were injected with a micro syringe pump. After staying for another 10 min, the syringe was slowly pulled out, and mice were placed on the insulation blanket until awakened. Tumor growth was verified and assessed by luciferase bioluminescence imaging at 1 and 21 days after implantation. The bioluminescence imaging was operated as follows: mice were anesthetized with isoflurane and injected with D‐luciferin (120 mg kg^−1^, i.p.), and bioluminescent signals in tumors were recorded 10 min later by using a Xenogen IVIS imaging system.

### In Silico Analysis

RNA‐seq reads were aligned to the reference genome in UCSC Genome Browser (http://genome.ucsc.edu/), which was used to visualize the STIM1 extra exon in the genomic context and explore the distribution of STIM1β. Cases were manually evaluated with the sequence alignment of the extra exon and its upstream and downstream exons. The normalized exon expression for each sample was generated by the RPKM method: RPKM = (reads x 109)/(total mapped read x exon length) and followed by log2 transformation.

The tertiary structure prediction was carried out using the I‐TASSER server (Iterative Threading ASSEmbly Refinement, https://zhanglab.ccmb.med.umich.edu/I‐TASSER/),^[^ ^39^ ^]^ which is an integrated platform for automated protein sequence‐to‐structure‐to‐function prediction server.

### Statistical Analysis

Quantitative data are shown mean ± sd or mean ± sem as indicated in the figure legends. The analyzed number (*n*) of samples and repeat times are listed for each experiment. Mice were randomly allocated to experimental groups. Acquired data were analyzed in Graphpad Prism 8 and Microsoft Excel 2013. The bar graph data were analyzed using paired or unpaired Student's *t*‐test. N.S. indicates no significance. *, **, ***, and **** represent *p* < 0.05, *p* < 0.01, *p* < 0.001, and *p* < 0.0001, respectively.

## Conflict of Interest

The authors declare no conflict of interest.

## Author Contributions

J.X., G.M., and L.Z. contributed equally to the work. Y.Z., G.M., Y.W., and W.H. conceived the ideas, designed the study, and directed the work. G.M., J.X., L.H., Z.H., T.W., Y.L., L.Z., L.W., and Y.W. designed and generated all the plasmid constructs. G.M., L.H., P.T., and Y.Z. characterized and analyzed STIM1β distribution. G.M., L.Z., J.L., Y.D., N.Z., Y.W., and Y.Z. explored the mechanism of STIM1β activation. X.L., G.M., X.L., R.W., Y.Z., and W.H. characterized STIM1β in vitro and in vivo experiments. L.W. performed patch‐clamp recordings. Z.Z. and L.H. contributed in silico analysis. W.H., S.S., S.F., S.W., and Y.H. provided intellectual input. G.M., X.L., and Y.Z. wrote the manuscript. L.Z., L.H., Z.Z., P.T., Z.H., S.F., T.W., Y.L., S.W., S.S., L.W., J.L., L.H., Y.H., R.W., Y.W., and W.H. reviewed and edited the manuscript.

## Supporting information

Supporting Information

Supplemental Movie 1

## Data Availability

The data that support the findings of this study are available from the corresponding author upon reasonable request.

## References

[1] a) M. Prakriya , R. S. Lewis , Physiol. Rev. 2015, 95, 1383. 26400989 10.1152/physrev.00020.2014 PMC4600950

[2] I. Derler , I. Jardin , C. Romanin , Am. J. Physiol. Cell Physiol. 2016, 310, C643. 26825122 10.1152/ajpcell.00007.2016 PMC4835918

[3] a) J. A. Rosado , R. Diez , T. Smani , I. Jardin , Front. Pharmacol. 2015, 6, 325. 26793113 10.3389/fphar.2015.00325 PMC4710697

[4] M. Trebak , J. P. Kinet , Nat. Rev. Immunol. 2019, 19, 154. 30622345 10.1038/s41577-018-0110-7 PMC6788797

[5] a) A. B. Parekh , Nat. Rev. Drug Discovery 2010, 9, 399. 20395953 10.1038/nrd3136

[6] G. R. Monteith , N. Prevarskaya , S. J. Roberts‐Thomson , Nat. Rev. Cancer 2017, 17, 367. 28386091 10.1038/nrc.2017.18

[7] a) A. F. Pla , K. Kondratska , N. Prevarskaya , Am. J. Physiol. Cell Physiol. 2016, 310, C509. 26791491 10.1152/ajpcell.00364.2015

[8] Y. Li , B. Guo , Q. Xie , D. Ye , D. Zhang , Y. Zhu , H. Chen , B. Zhu , Cell Rep. 2015, 12, 388. 26166565 10.1016/j.celrep.2015.06.033

[9] K. J. Wood , A. Bushell , J. Hester , Nat. Rev. Immunol. 2012, 12, 417. 22627860 10.1038/nri3227

[10] S. Feske , Ann. N. Y. Acad. Sci. 2011, 1238, 74. 22129055 10.1111/j.1749-6632.2011.06240.x PMC3774594

[11] C. Neftel , J. Laffy , M. G. Filbin , T. Hara , M. E. Shore , G. J. Rahme , A. R. Richman , D. Silverbush , M. L. Shaw , C. M. Hebert , J. Dewitt , S. Gritsch , E. M. Perez , L. N. G. Castro , X. Lan , N. Druck , C. Rodman , D. Dionne , A. Kaplan , M. S. Bertalan , J. Small , K. Pelton , S. Becker , D. Bonal , Q. D. Nguyen , R. L. Servis , J. M. Fung , R. Mylvaganam , L. Mayr , J. Gojo , et al., Cell 2019, 178, 835. 31327527 10.1016/j.cell.2019.06.024 PMC6703186

[12] H. Liu , J. D. Hughes , S. Rollins , B. Chen , E. Perkins , Exp. Mol. Pathol. 2011, 91, 753. 21945734 10.1016/j.yexmp.2011.09.005

[13] R. K. Motiani , M. C. Hyzinski‐Garcia , X. Zhang , M. M. Henkel , I. F. Abdullaev , Y. H. Kuo , K. Matrougui , A. A. Mongin , M. Trebak , Pfluegers Arch. ‐ Eur. J. Physiol. 2013, 465, 1249. 23515871 10.1007/s00424-013-1254-8 PMC3748246

[14] G. Li , Z. Zhang , R. Wang , W. Ma , Y. Yang , J. Wei , Y. Wei , J. Exp. Clin. Cancer Res. 2013, 32, 20. 23578185 10.1186/1756-9966-32-20 PMC3639102

[15] Z. Liu , H. Li , L. He , Y. Xiang , C. Tian , C. Li , P. Tan , J. Jing , Y. Tian , L. Du , Y. Huang , L. Han , M. Li , Y. Zhou , Cell Chem. Biol. 2019, 26, 352. 30639261 10.1016/j.chembiol.2018.11.009 PMC6430684

[16] F. Michelangeli , J. M. East , Biochem. Soc. Trans. 2011, 39, 789. 21599650 10.1042/BST0390789

[17] G. Ma , Q. Zhang , L. He , N. T. Nguyen , S. Liu , Z. Gong , Y. Huang , Y. Zhou , Chem. Sci. 2018, 9, 5551. 30061986 10.1039/c8sc00839f PMC6048692

[18] I. Grigoriev , S. M. Gouveia , B. van der Vaart , J. Demmers , J. T. Smyth , S. Honnappa , D. Splinter , M. O. Steinmetz , J. W. Putney, Jr. , C. C. Hoogenraad , A. Akhmanova , Curr. Biol. 2008, 18, 177. 18249114 10.1016/j.cub.2007.12.050 PMC2600655

[19] G. Ma , M. Wei , L. He , C. Liu , B. Wu , S. L. Zhang , J. Jing , X. Liang , A. Senes , P. Tan , S. Li , A. Sun , Y. Bi , L. Zhong , H. Si , Y. Shen , M. Li , M. S. Lee , W. Zhou , J. Wang , Y. Wang , Y. Zhou , Nat. Commun. 2015, 6, 7826. 26184105 10.1038/ncomms8826 PMC4509486

[20] a) M. Muik , M. Fahrner , I. Derler , R. Schindl , J. Bergsmann , I. Frischauf , K. Groschner , C. Romanin , J. Biol. Chem. 2009, 284, 8421. 19189966 10.1074/jbc.C800229200 PMC2659200

[21] G. Ma , L. He , S. Liu , J. Xie , Z. Huang , J. Jing , Y. T. Lee , R. Wang , H. Luo , W. Han , Y. Huang , Y. Zhou , Nat. Commun. 2020, 11, 1039. 32098964 10.1038/s41467-020-14841-9 PMC7042325

[22] S. Zheng , G. Ma , L. He , T. Zhang , J. Li , X. Yuan , N. T. Nguyen , Y. Huang , X. Zhang , P. Gao , R. Nwokonko , D. L. Gill , H. Dong , Y. Zhou , Y. Wang , PLoS Biol. 2018, 16, e2006898. 30444880 10.1371/journal.pbio.2006898 PMC6267984

[23] T. Kawasaki , I. Lange , S. Feske , Biochem. Biophys. Res. Commun. 2009, 385, 49. 19433061 10.1016/j.bbrc.2009.05.020 PMC2821023

[24] A. Jha , M. Ahuja , J. Maleth , C. M. Moreno , J. P. Yuan , M. S. Kim , S. Muallem , J. Cell Biol. 2013, 202, 71. 23816623 10.1083/jcb.201301148 PMC3704993

[25] H. Ruiz‐Garcia , K. Alvarado‐Estrada , P. Schiapparelli , A. Quinones‐Hinojosa , D. M. Trifiletti , Front. Cell. Neurosci. 2020, 14, 558381. 33177991 10.3389/fncel.2020.558381 PMC7596188

[26] F. E. Baralle , J. Giudice , Nat. Rev. Mol. Cell Biol. 2017, 18, 437. 28488700 10.1038/nrm.2017.27 PMC6839889

[27] S. C. Bonnal , I. Lopez‐Oreja , J. Valcarcel , Nat. Rev. Clin. Oncol. 2020, 17, 457. 32303702 10.1038/s41571-020-0350-x

[28] M. L. Knapp , K. Förderer , D. Alansary , M. J. Yvonne Schwarz , A. Lis , B. A. Niemeyer , bioRxiv 2020, 2020.03.25.005199.

[29] L. Zheng , P. B. Stathopulos , R. Schindl , G. Y. Li , C. Romanin , M. Ikura , Proc. Natl. Acad. Sci. USA 2011, 108, 1337. 21217057 10.1073/pnas.1015125108 PMC3029719

[30] M. Fahrner , M. Muik , R. Schindl , C. Butorac , P. Stathopulos , L. Zheng , I. Jardin , M. Ikura , C. Romanin , J. Biol. Chem. 2014, 289, 33231. 25342749 10.1074/jbc.M114.610022 PMC4246082

[31] C. Bertoli , J. M. Skotheim , R. A. de Bruin , Nat. Rev. Mol. Cell Biol. 2013, 14, 518. 23877564 10.1038/nrm3629 PMC4569015

[32] H. Zhao , G. Yan , L. Zheng , Y. Zhou , H. Sheng , L. Wu , Q. Zhang , J. Lei , J. Zhang , R. Xin , L. Jiang , X. Zhang , Y. Chen , J. Wang , Y. Xu , D. Li , Y. Li , Theranostics 2020, 10, 6483. 32483465 10.7150/thno.44025 PMC7255033

[33] Y. F. Chen , W. T. Chiu , Y. T. Chen , P. Y. Lin , H. J. Huang , C. Y. Chou , H. C. Chang , M. J. Tang , M. R. Shen , Proc. Natl. Acad. Sci. USA 2011, 108, 15225. 21876174 10.1073/pnas.1103315108 PMC3174613

[34] C. Dubois , F. Vanden Abeele , V. Lehen'kyi , D. Gkika , B. Guarmit , G. Lepage , C. Slomianny , A. S. Borowiec , G. Bidaux , M. Benahmed , Y. Shuba , N. Prevarskaya , Cancer Cell 2014, 26, 19. 24954132 10.1016/j.ccr.2014.04.025

[35] K. P. Subedi , H. L. Ong , G. Y. Son , X. Liu , I. S. Ambudkar , Cell Rep. 2018, 23, 522. 29642009 10.1016/j.celrep.2018.03.065

[36] S. Zheng , L. Zhou , G. Ma , T. Zhang , J. Liu , J. Li , N. T. Nguyen , X. Zhang , W. Li , R. Nwokonko , Y. Zhou , F. Zhao , J. Liu , Y. Huang , D. L. Gill , Y. Wang , Pfluegers Arch. ‐ Eur. J. Physiol. 2018, 470, 1555. 29934936 10.1007/s00424-018-2165-5 PMC6153602

[37] Y. Zhao , S. Araki , J. Wu , T. Teramoto , Y. F. Chang , M. Nakano , A. S. Abdelfattah , M. Fujiwara , T. Ishihara , T. Nagai , R. E. Campbell , Science 2011, 333, 1888. 21903779 10.1126/science.1208592 PMC3560286

[38] L. He , L. Wang , H. Zeng , P. Tan , G. Ma , S. Zheng , Y. Li , L. Sun , F. Dou , S. Siwko , Y. Huang , Y. Wang , Y. Zhou , Nat. Commun. 2021, 12, 164. 33431868 10.1038/s41467-020-20425-4 PMC7801460

[39] J. Yang , R. Yan , A. Roy , D. Xu , J. Poisson , Y. Zhang , Nat. Methods 2015, 12, 7. 25549265 10.1038/nmeth.3213 PMC4428668

