# Supplementary material for: Identification of a STIM1 Splicing Variant that Promotes Glioblastoma Growth
Source: Adv Sci (Weinh). 2022 Jan 25;9(11):2103940. doi: 10.1002/advs.202103940 (PMC9008427; doi:10.1002/advs.202103940)
Supplement: Supplementary file 1 — Supporting Information [file ADVS-9-2103940-s001.pdf]

ADVANCED SCIENCE
Open Access

Supporting Information

for *Adv. Sci.*, DOI 10.1002/advs.202103940

Identification of a STIM1 Splicing Variant that Promotes Glioblastoma Growth

Jiansheng Xie, Guolin Ma, Lijuan Zhou, Lian He, Zhao Zhang, Peng Tan, Zixian Huang, Shaohai Fang, Tianlu Wang, Yi-Tsang Lee, Shufan Wen, Stefan Siwko, Liuqing Wang, Jindou Liu, Yangchun Du, Ningxia Zhang, Xiaoxuan Liu, Leng Han, Yun Huang, Rui Wang, Youjun Wang*, Yubin Zhou* and Weidong Han**

ADVANCED SCIENCE
Open Access

Supporting Information

for *Adv. Sci.*, DOI: 10.1002/advs.202103940

Identification of a STIM1 Splicing Variant that Promotes Glioblastoma Growth

Jiansheng Xie[#,1], Guolin Ma[#,,2], Lijuan Zhou[#,3], Lian He[2], Zhao Zhang[4,5], Peng Tan[2], Zixian Huang[2], Shaohai Fang[6], Tianlu Wang[2], Yi-Tsang Lee[2], Shufan Wen[2], Stefan Siwko[2], Liuqing Wang[3], Jindou Liu[3], Yangchun Du[3], Ningxia Zhang[1], Xiaoxuan Liu[2], Leng Han[5,6], Yun Huang[6], Rui Wang[2], Youjun Wang*[,3], Yubin Zhou*[,2,7], Weidong Han*[,1]*

Supporting information for

Identification of a STIM1 Splicing Variant that Promotes Glioblastoma Growth

Contents:

Supplementary Figures 1-13

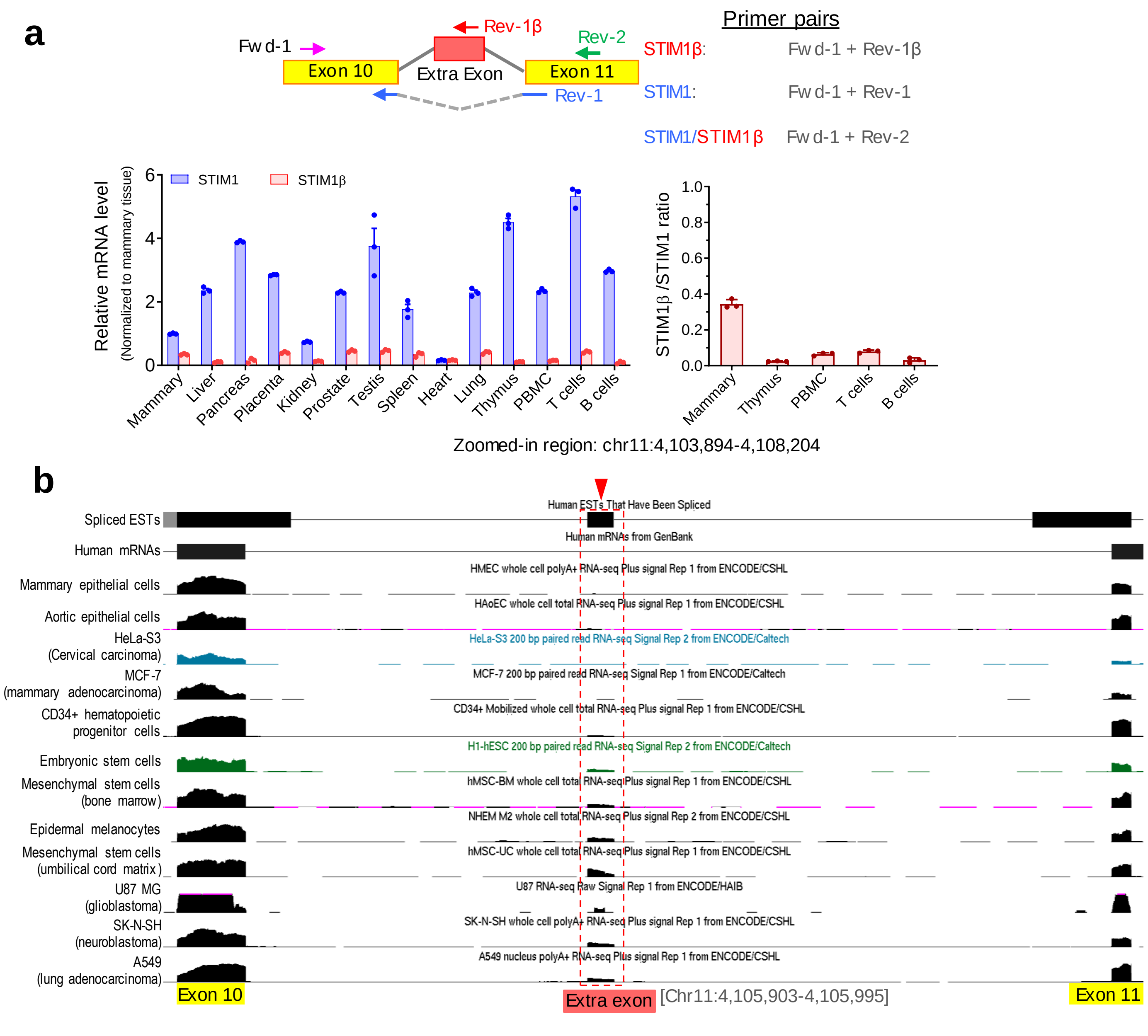

Supplementary Figure 1 | Tissue distribution of an alternative splicing variant of STIM1 (STIM1β)

(**a**) Upper panel: schematic representation of an alternative splicing variant and primer pairs used in qRT-PCR analysis. Bottom panel: relative quantities of STIM1 and STIM1β in a panel of human tissues, expressed as the relative mRNA level (normalized to mammary tissue, left), and the ratio of STIM1β/STIM1 in selected tissues (right). The relative expression level of STIM1 or STIM1β was normalized to STIM1 level in the mammary tissue

(**b**) Zoomed-in regions of UCSC Genome Browser on Human Feb. 2009 (GRCh37/hg19)): chr11:4,103,894-4,108,204. The extra exon was highlighted by a red rectangle and exons 10/11 were highlighted in yellow.

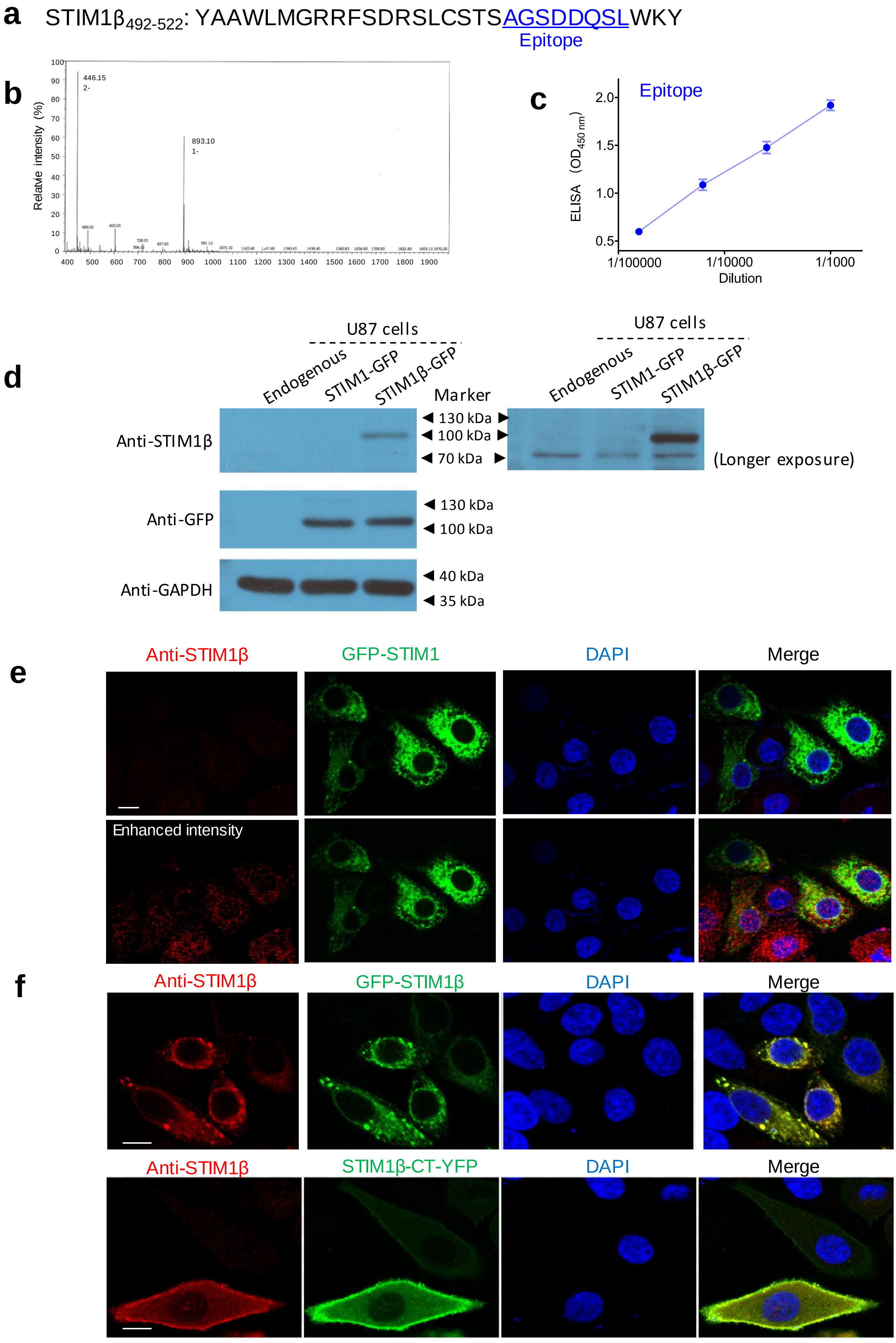
a
STIM1β492-522: YAAWLMGRRFSDRSLCSTSAGSDDQSLWKY
Epitope
b
Relatvie intensity (%)
446.15
2-
893.10
1-
c
Epitope
ELISA (OD450 nm)
1/100000
1/10000
1/1000
Dilution
d
U87 cells
Endogenous
STIM1-GFP
STIM1β-GFP
Marker
Anti-STIM1β
130 kDa
100 kDa
70 kDa
(Longer exposure)
Anti-GFP
Anti-GAPDH
40 kDa
35 kDa
e
Anti-STIM1β
GFP-STIM1
DAPI
Merge
Enhanced intensity
f
GFP-STIM1β
STIM1β-CT-YFP

Supplementary Figure 2 | Generation and characterization of an antibody specifically against STIM1β.

(**a**) The epitope selected in the STIM1β-PAD region for antibody production.

(**b**) Confirmation of the synthesized peptide as epitope by mass spectrometry.

(**c**) ELISA evaluation of the sensitivity of the anti-sera obtained from a rabbit immunized with the STIM1β peptide.

(**d**) Testing the specificity of the generated antibody. U87 cells and U87 cells overexpressing GFP-tagged STIM1 and STIM1β constructs were lysed and analyzed with the home-made antibody or the anti-GFP antibody to probe anti-STIM1β, anti-STIM1, and anti-GFP, respectively. GAPDH was used as a loading control.

(**e-f**) Fluorescent immunostaining evaluation of STIM1β protein levels in HeLa cells expressing GFP-STIM1 (**e**), GFP-STIM1β (**f**, top panel) and STIMβ$_{233\text{-}716}$-(or STIM1β-CT)-YFP (**f**, bottom panel). An anti-rabbit Alexa 568 secondary antibody (red) and nuclear DAPI staining (blue) were used to visualize STIM1β or STIM1β-CT and the nuclei, respectively. In panel e (bottom), the red channel was over-exposed (5 times longer than the top panels) to indicate the shape of the cells, and also to demonstrate no unspecific binding of the anti-STIM1β antibody toward wild-type GFP-STIM1. Scale bar 10 µm.

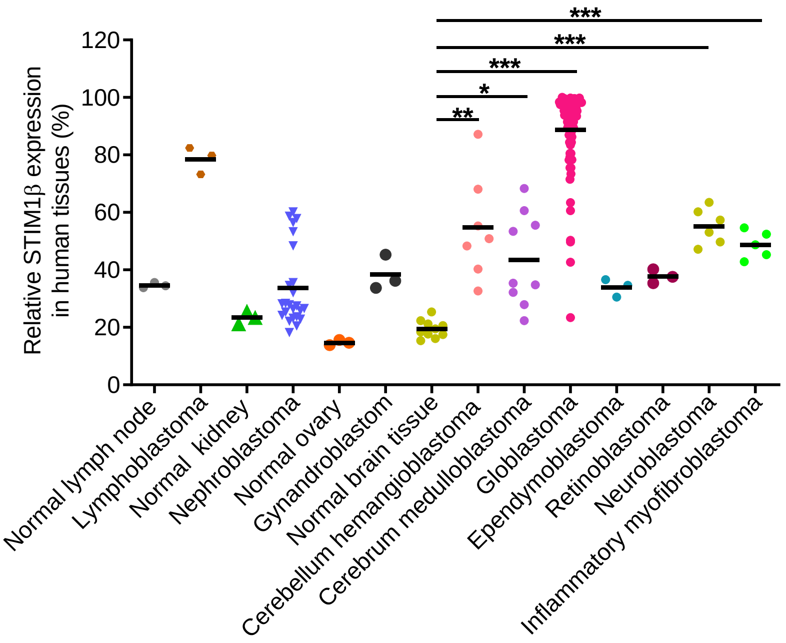

Supplementary Figure 3 | Analysis of STIM1β expression in normal and cancer tissues.
Quantitative assessment of STIM1β expression using IHC staining in paraffin-based tissue array, which contains 80 patient samples covering both normal and cancer tissues. The relative expression of STIM1β was normalized against the sample with the highest immunostaining intensity.

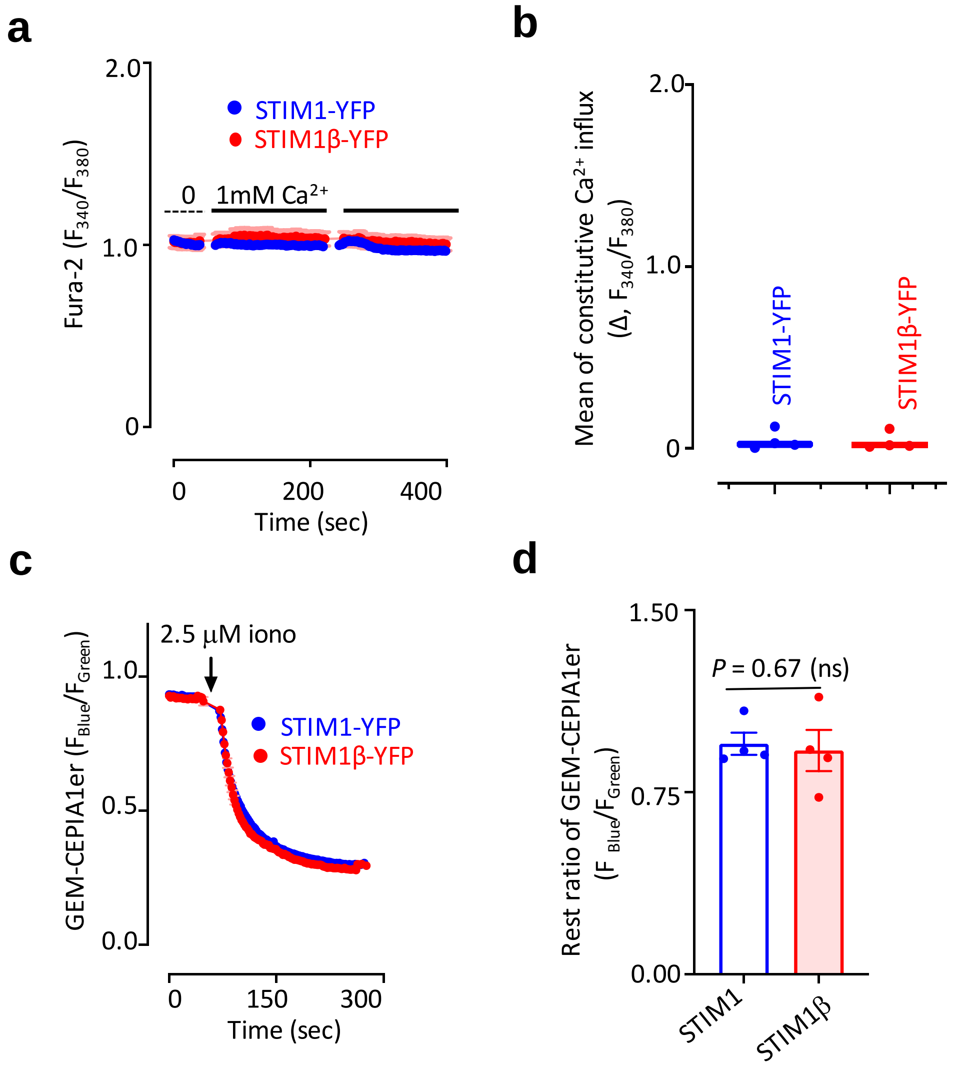

Supplementary Figure 4 | Evaluating the cytosolic Ca^{2+} and ER Ca^{2+} store in HEK293 cells expressing STIM1-YFP or STIM1β-YFP.

(**a**) Cytosolic Ca^{2+} was indicated by Fura-2 ratio in HEK293 cells transfected with STIM1-YFP or STIM1β-YFP, respectively, at comparable levels. Constitutive Ca^{2+} entry was evaluated by switching the external media between Ca^{2+} free (0 Ca^{2+}) and Ca^{2+}-loading buffers (1 mM Ca^{2+}).

(**b**) Statistics of the mean constitutive Ca^{2+} influx indicated by changes in Fura-2 ratio induced by 1 mM Ca^{2+}. n = 4, 30-50 cells from each repeat. Data were shown as mean ± sem.

(**c**-**d**) Analysis of the ER luminal Ca^{2+} level in HEK GEM-CEPIA1er stable cells transfected with STIM1-YFP or STIM1β-YFP. The store depletion was triggered by 2.5 µM ionomycin. (**c**) Representative traces of ER luminal Ca^{2+} response (STIM1, n = 66 cells; STIM1β, n = 47 cells) . (**d**) Quantification of the signal of GEM-CEPIA1er at the resting condition. Each dot represented the averaged value of 30 ~ 90 cells from four biological replicates. Data were shown as mean ± sem (unpaired Student's *t*-test).

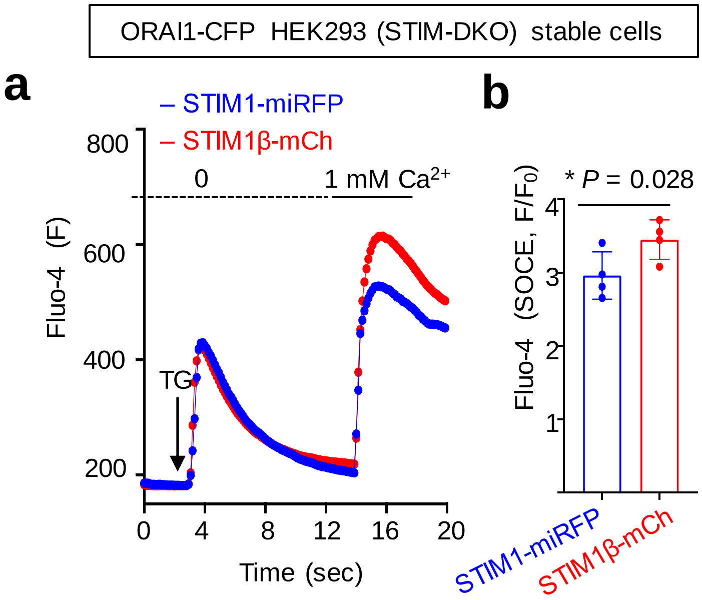

Supplementary Figure 5 | Side-by-side comparison of the contribution of STIM1 or STIM1β to SOCE under the same imaging field.

(**a**) Ca^{2+} influx was monitored by Fluo-4 fluorescence in ORAI1-CFP expressing HEK293 stable cells with STIM-DKO and co-transfected with STIM1-miRFP and STIM1β-mCh. The plasmids of STIM1-miRFP and STIM1β-mCh were prepared in the same vector using the same linker. Plasmids encoding STIM1 and STIM1β were individually transfected into ORAI-CFP HEK293with a STIM1/STIM2 double-knockout (STIM-DKO) background. 6 hours after transfection, cells were merged into the same dish. 12 hours later, Ca^{2+} imaging was performed using Fluo4-AM. Shown were representative traces of SOCE response upon TG treatment and Ca^{2+} add-back (left, n = 45 cells). Store depletion was induced by 1 µM TG.

(**b**) The mean amplitude of SOCE (n = 4) was summarized on the bar graph. Each dot represents the averaged value of 30 ~ 60 cells. Data were shown as mean ± sem (Paired Student's *t*-test).

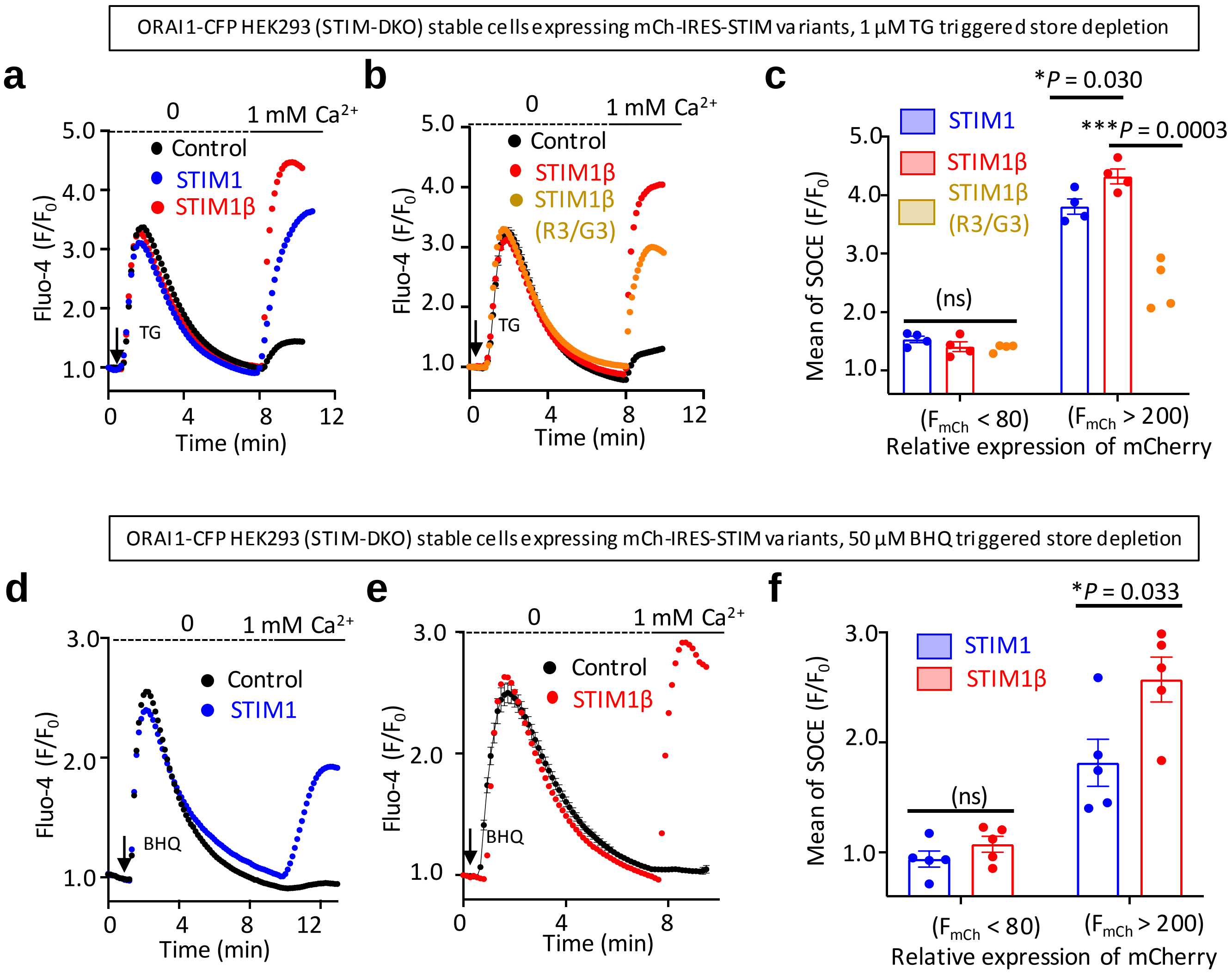

Supplementary Figure 6 | Quantitative analysis of SOCE responses in the indicated cells expressing STIM1 or STIM1β with different ER Ca^{2+} store depletion reagents.

(**a**) Representative mean intracellular Ca^{2+} traces of ORAI-CFP HEK293 STIM-DKO cells expressing mCh-IRES-STIM1 (n = 81) or mCh-IRES-STIM1β (n = 48). The expression level (F) of mCherry (mCh) was used to indicate and separate HEK STIM-DKO control cells (F < 80, n = 39) and STIM-expressing cells (F > 200) in the same imaging field. Fluo-4 was used to monitor Ca^{2+} influx. ER Ca^{2+} store depletion was induced by 1 µM TG.

(**b**) Representative mean intracellular Ca^{2+} traces of ORAI-CFP HEK293 STIM-DKO cells expressing mCh-IRES-STIM1β (n = 40) or its mutant R3/G3 (R499G/R500G/R504G; n = 61 cells). n = 32 cells for the control group. ER Ca^{2+} store depletion was induced by 1 µM TG.

(**c**) Quantification of SOCE amplitudes triggered by 1 µM TG in ORAI-CFP HEK293 STIM-DKO cells (control; $F_{mCh} < 80$) and cells expressing STIM1, STIM1β, or STIM1β (R3/G3) mutant. n = 4 independent biological replicates. Each dot represented the average value of SOCE from 30 ~ 120 cells.

(**d-e**) Representative mean intracellular Ca^{2+} traces of ORAI-CFP HEK293 STIM-DKO cells expressing mCh-IRES-STIM1 (**d**) (n = 120) or mCh-IRES-STIM1β (**e**, n = 114). Fluo-4 was used to monitor Ca^{2+} influx. ER Ca^{2+} store depletion was induced by 50 µM BHQ.

(**f**) Graph showing the mean amplitudes of SOCE after treatment of BHQ (n = 5), obtained by measuring the increase of Fluo-4 fluorescence at the SOCE peak compared to the baseline. Each dot represented the average value from 30 ~ 120 cells. All data were shown as mean ± sem (Unpaired Student's *t*-test).

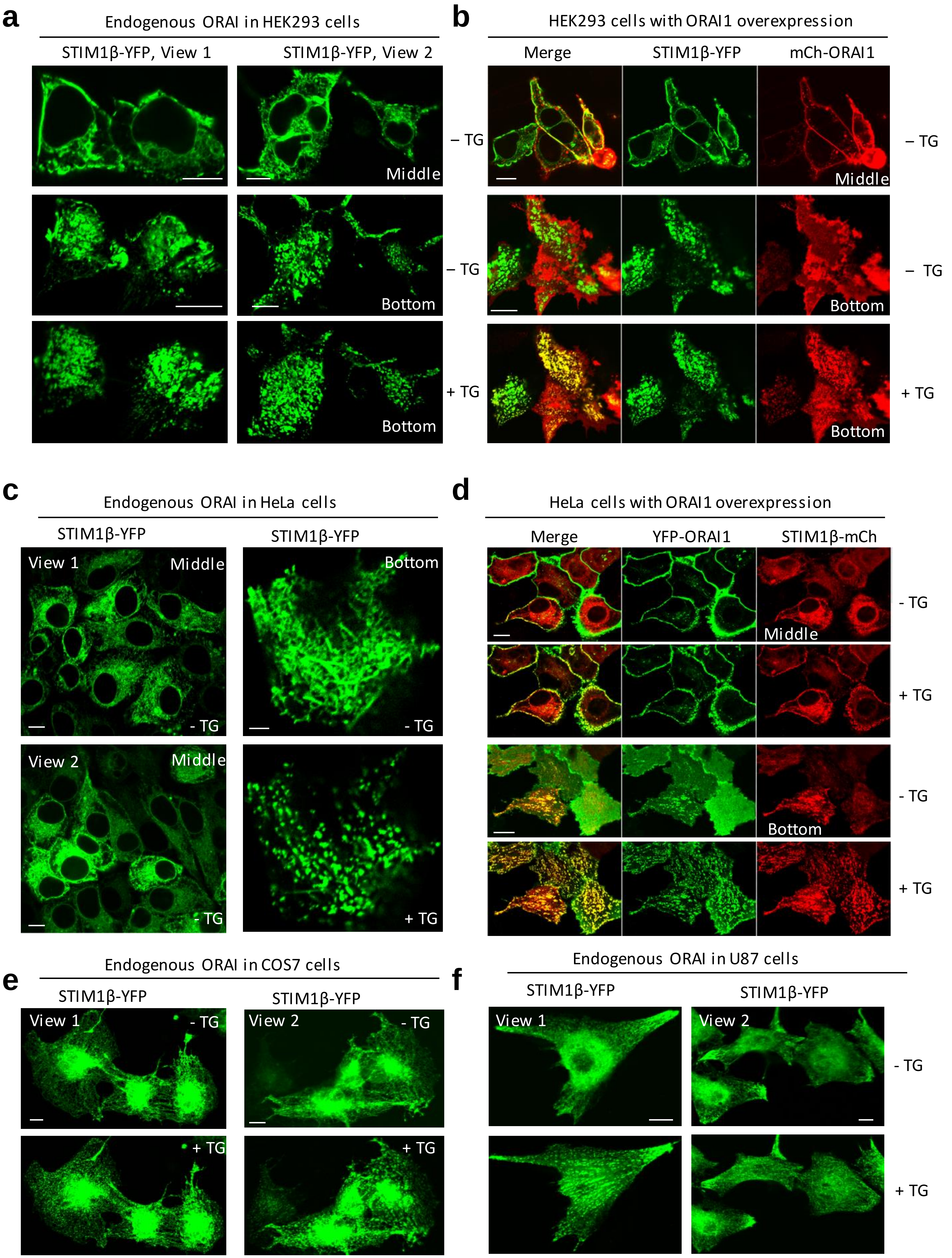
a
Endogenous ORAI in HEK293 cells
STIM1β-YFP, View 1
STIM1β-YFP, View 2
Middle
Bottom
Bottom
– TG
– TG
+ TG
b
HEK293 cells with ORAI1 overexpression
Merge
STIM1β-YFP
mCh-ORAI1
Middle
Bottom
Bottom
– TG
– TG
+ TG
c
Endogenous ORAI in HeLa cells
STIM1β-YFP
STIM1β-YFP
View 1
Middle
Bottom
- TG
- TG
View 2
Middle
- TG
+ TG
d
HeLa cells with ORAI1 overexpression
Merge
YFP-ORAI1
STIM1β-mCh
Middle
Bottom
- TG
+ TG
- TG
+ TG
e
Endogenous ORAI in COS7 cells
STIM1β-YFP
STIM1β-YFP
View 1
- TG
View 2
- TG
+ TG
+ TG
f
Endogenous ORAI in U87 cells
STIM1β-YFP
STIM1β-YFP
View 1
View 2
- TG
+ TG

Supplementary Figure 7 | Characterization of STIM1β puncta formation in various cells.

(**a**) Selected confocal images of HEK293 cells transfected with STIM1β-YFP before and after addition of 1 μM TG. Two views were selected. A portion of STIM1β-YFP spontaneously formed puncta in the absence of TG, indicating a propensity of STIM1β-YFP toward pre-activation.

(**b**) Confocal images showing that most STIM1β-YFP constitutively formed puncta with overexpressed mCh-ORAI1 in HEK293 cells without store depletion.

(**c**) Confocal images showing that STIM1β-YFP evenly distributed throughout the ER network, indicating that STIM1β adopted a largely inactive conformation (left, view 1 and view 2). Right panel, footprint images of STIM1β-YFP forming puncta after TG-triggered store depletion.

(**d**) Selected middle and bottom views of HeLa cells co-expressing STIM1β-YFP and mCh-ORAI1 before and after TG stimulation.

(**e**-**f**) Multiple views from confocal images of COS-7 (**e**) and U87 cells (**f**) expressing STIM1β-YFP before and after TG-induced store depletion. Scale bar, 10 μm.

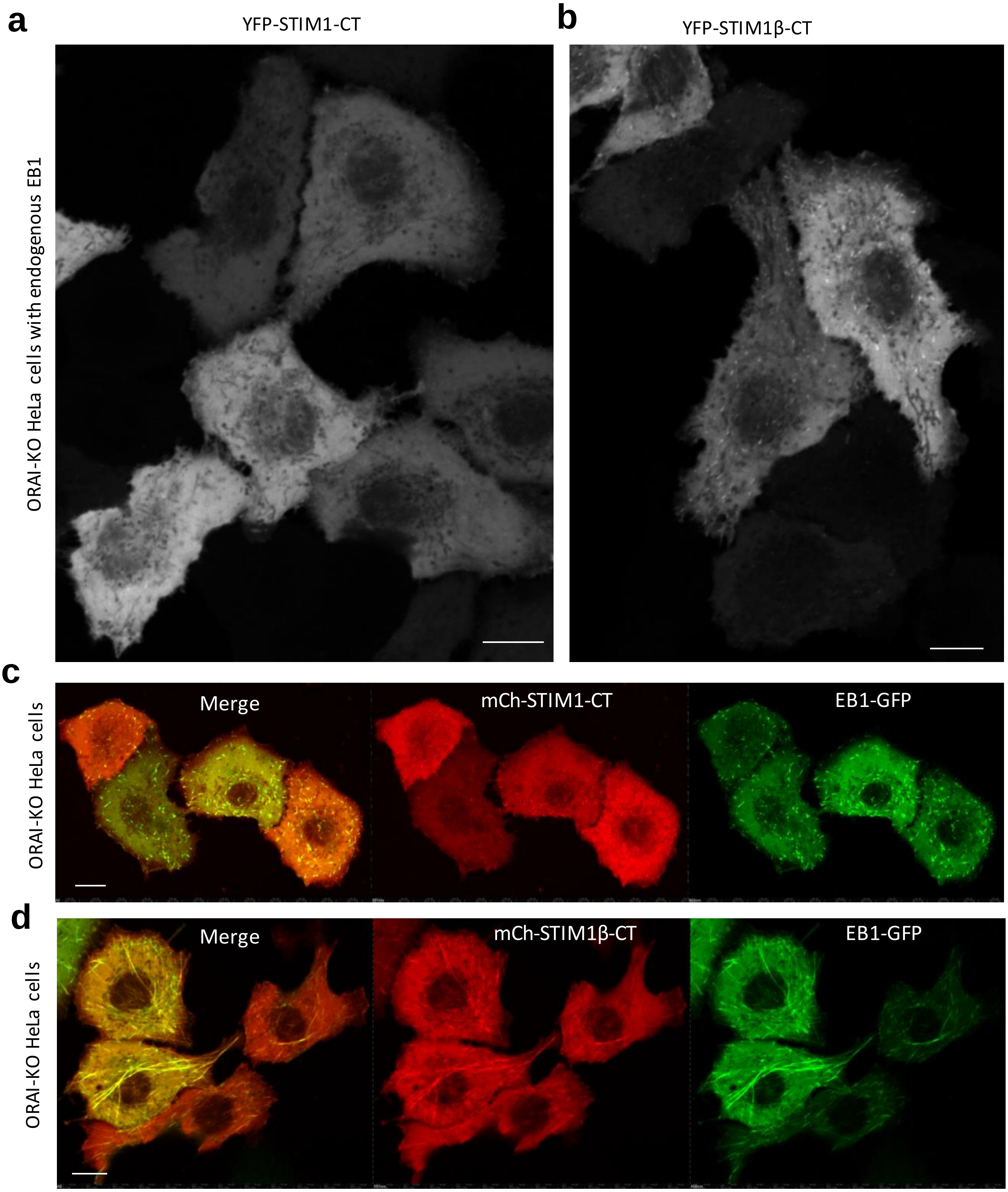

Supplementary Figure 8| Patterns of microtubule plus ending tracking mediated by STIM1-CT or STIM1β-CT.
(**a**-**b**) Typical confocal images of ORAI-KO HeLa cells expressing YFP-STIM1-CT (**a**) or YFP-STIM1β-CT (**b**).
(**c**-**d**) Confocal images showing stronger colocalization of mCh-STIM1β-CT with EB1-GFP, when compared to mCh-STIM1-CT, in ORAI-KO HeLa cells. Cells were co-transfected with EB1-GFP + mCh-STIM1β-CT (**c**) or EB1-GFP + mCh-STIM1β-CT (**d**), respectively. The pattern of microtubule plus end (+TIPs) tracking can be used to discriminate the protein oligomeric states using a fluorescence microscope. Because STIM1β-CT may exist as a high-order oligomer and adopts a partially activated conformation, STIM1β-CT displayed comet-like patterns, tracking along MT plus ends (+TIPs) when compared to STIM1-CT (**a**). Co-expression with EB1-GFP promoted +TIPs tracking (**c**-**d**), with STIM1β-CT displaying notable MT binding and the appearance of visible tubular structures (**d**). Scale bar, 10 μm.

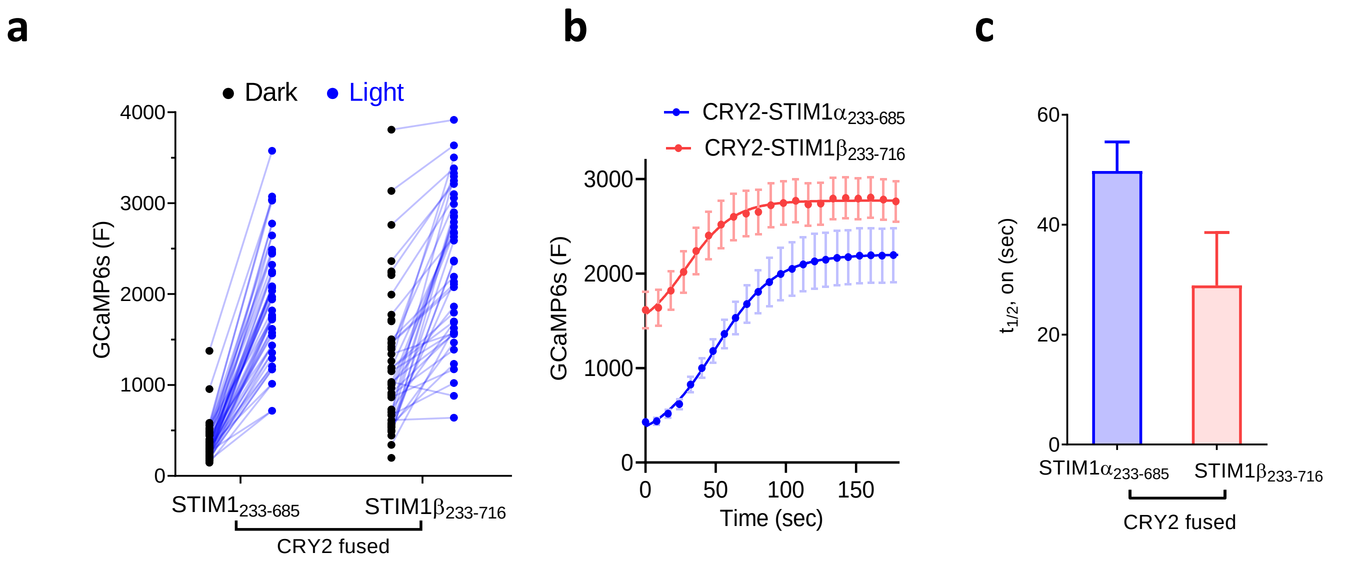

Supplementary Figure 9 | Light-inducible Ca^{2+} influx mediated by CRY2-fused STIM1β-CT fragments.
(**a**) Comparison of cytosolic Ca^{2+} levels reported by GCaMP6s in HeLa cells expressing mCh-CRY2-STIM1$_{233\text{-}685}$ and mCh-CRY2-STIM1β$_{233\text{-}716}$ before and after blue light illumination at 470 nm. Some cells transfected with mCh-CRY2-STIM1β$_{233\text{-}716}$ showed pre-activation with higher basal GCaMP6s signals in the dark.
(**b**) Time course of light-induced Ca^{2+} influx in HeLa cells expressing the indicated hybrid constructs.
(**c**) Comparison of the activation half-lives of CRY2-STIM1$_{233\text{-}685}$ and CRY2-STIM1β$_{233\text{-}716}$. n = 50 cells. Data are shown as mean ± sem.

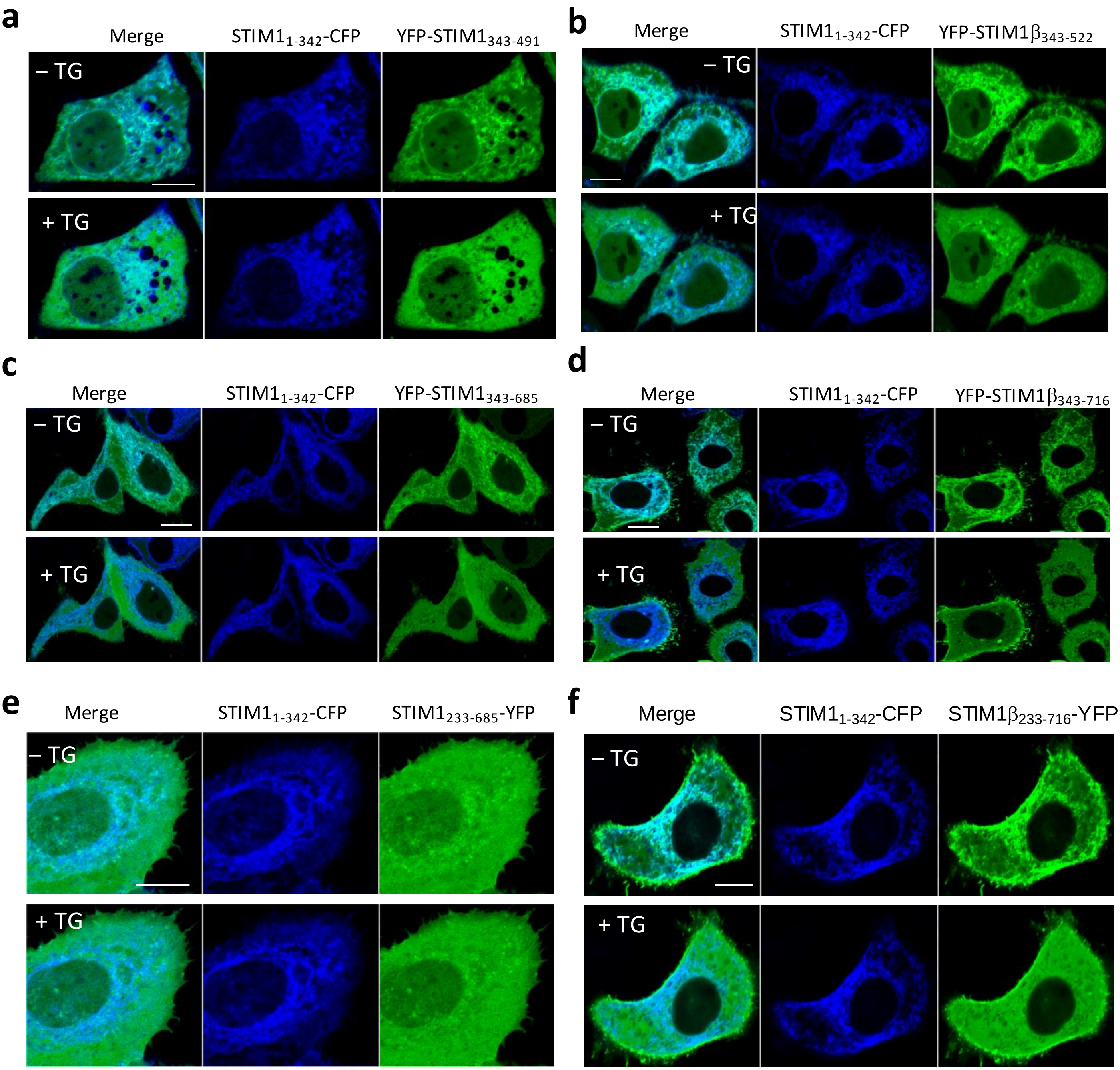

Supplementary Figure 10 | Comparison of the co-localization between CC1 and STIM1 cytoplasmic fragments derived from STIM1 and STIM1β.

(a-f) Representative confocal images of HeLa cells co-transfected with STIM1$_{1-342}$-CFP and YFP tagged various STIM1 or STIM1β cytoplasmic fragments: (a-b) STIM1$_{343-491}$ *vs* STIM1β$_{343-522}$; (c-d) STIM1$_{343-685}$ *vs* STIM1β$_{343-716}$, (e-f) STIM1$_{233-685}$ *vs* STIM1β$_{233-716}$. Scale bar, 10 μm.

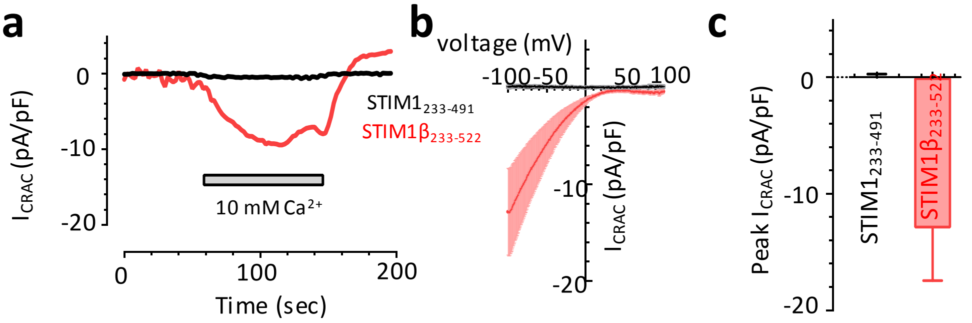

Supplementary Figure 11 | Electrophysiological characterization in cells with the indicated conditions.

(**a**) Typical time course of I_{CRAC} current density of HEK ORAI1-CFP cells expressing YFP-STIM1$_{233\text{-}491}$ (black) and YFP-STIM1β$_{233\text{-}522}$ (red) in the presence of 10 mM Ca^{2+}.

(**b**) Mean I-V relationships at the peak of CRAC currents.

(**c**) Quantification of peak I_{CRAC}. n = 6-7 cells. Data are shown as mean ± sem.

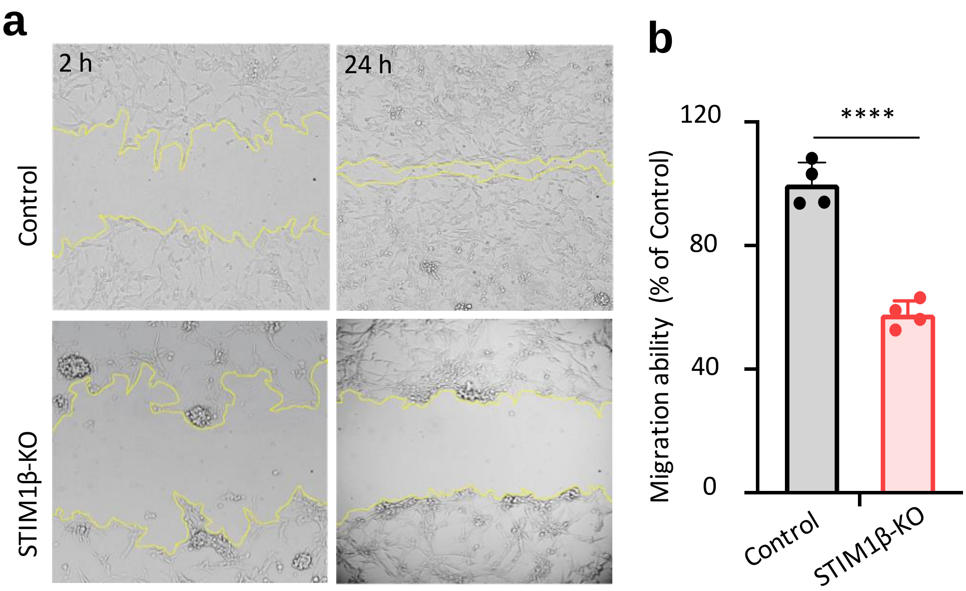

Supplementary Figure 12 | Wound healing assay to assess the effect of STIM1β-KO on U87 cell migration.
(**a**) Representative wound healing images. The boundaries of open wound regions were highlighted as yellow contours by Image J.
(**b**) Quantification of the migration ability for U87 control and STIM1β-KO cells (n=4). The relative changes in the calculated areas of gap regions was used to indicate the migration ability. **** *P* < 0.0001. Unpaired Student's t-test.

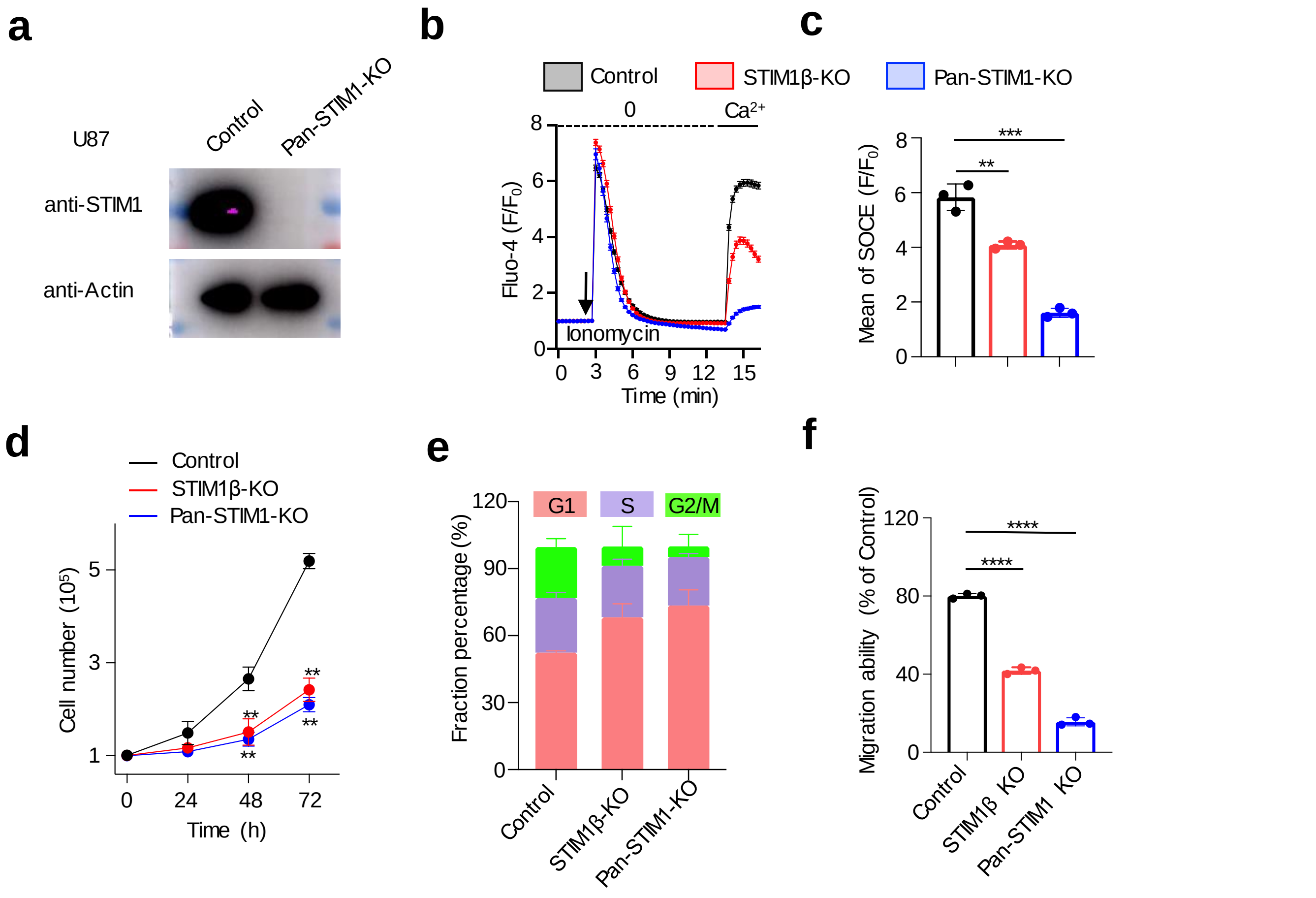

Supplementary Figure 13 | Comparing the effects of pan-STIM1 (both conventional STIM1 and STIM1β) depletion versus STIM1β-KO on SOCE, growth and invasion of U87 GBM cells.

(**a**) Confirmation of pan-STIM1 knockout in U87 cells by immunoblotting. An anti-STIM1 antibody (#610954, BD Bioscience) that recognizes the N-terminal $STIM_{125\text{-}195}$ shared by both conventional STIM1 and STIM1β was used.

(**b**) Representative traces of cytosolic Ca^{2+} response to store depletion triggered by 5 µM ionomycin in WT, STIM1β-KO, or pan-STIM1-KO U87 cells.

(**c**) Quantification of mean SOCE responses in the indicated groups (n=3; 30~60 cells were selected for each repeat).

(**d**-**f**) Effects of STIM1 knockout on U87 cell viability (n=3) (d), cell cycle (n=2) (e), and cell migration (n=3) (f).

Data are shown as mean ± sem. $^{**}P < 0.01$, $^{***}P < 0.001$, $^{****}P < 0.0001$. Unpaired Student's t-test.
